# Identification of molecular targets and small drug candidates for Huntington's disease via bioinformatics and a network‐based screening approach

**DOI:** 10.1111/jcmm.18588

**Published:** 2024-08-17

**Authors:** Md Ridoy Hossain, Md. Mohaimenul Islam Tareq, Partha Biswas, Sadia Jannat Tauhida, Shabana Bibi, Md. Nazmul Hasan Zilani, Ghadeer M. Albadrani, Muath Q. Al‐Ghadi, Mohamed M. Abdel‐Daim, Md. Nazmul Hasan

**Affiliations:** ^1^ Laboratory of Pharmaceutical Biotechnology and Bioinformatics, Department of Genetic Engineering and Biotechnology Jashore University of Science and Technology Jessore Bangladesh; ^2^ Department of Biosciences Shifa Tameer‐e‐Millat University Islamabad Pakistan; ^3^ Department of Health Sciences Novel Global Community Educational Foundation Hebersham New South Wales Australia; ^4^ Department of Pharmacy Jashore University of Science and Technology Jessore Bangladesh; ^5^ Department of Biology, College of Science Princess Nourah bint Abdulrahman University Riyadh Saudi Arabia; ^6^ Department of Zoology, College of Science King Saud University Riyadh Saudi Arabia; ^7^ Department of Pharmaceutical Sciences, Pharmacy Program Batterjee Medical College Jeddah Saudi Arabia; ^8^ Pharmacology Department, Faculty of Veterinary Medicine Suez Canal University Ismailia Egypt

**Keywords:** CAG (cytosine–adenine–guanine), degenerative, gene set enrichment analysis (GSEA), Huntington's disease

## Abstract

Huntington's disease (HD) is a gradually severe neurodegenerative ailment characterised by an increase of a specific trinucleotide repeat sequence (cytosine–adenine–guanine, CAG). It is passed down as a dominant characteristic that worsens over time, creating a significant risk. Despite being monogenetic, the underlying mechanisms as well as biomarkers remain poorly understood. Furthermore, early detection of HD is challenging, and the available diagnostic procedures have low precision and accuracy. The research was conducted to provide knowledge of the biomarkers, pathways and therapeutic targets involved in the molecular processes of HD using informatic based analysis and applying network‐based systems biology approaches. The gene expression profile datasets GSE97100 and GSE74201 relevant to HD were studied. As a consequence, 46 differentially expressed genes (DEGs) were identified. 10 hub genes (*TPM1*, *EIF2S3*, *CCN2*, *ACTN1*, *ACTG2*, *CCN1*, *CSRP1*, *EIF1AX*, *BEX2* and *TCEAL5*) were further differentiated in the protein–protein interaction (PPI) network. These hub genes were typically down‐regulated. Additionally, DEGs‐transcription factors (TFs) connections (e.g. GATA2, YY1 and FOXC1), DEG‐microRNA (miRNA) interactions (e.g. hsa‐miR‐124‐3p and has‐miR‐26b‐5p) were also comprehensively forecast. Additionally, related gene ontology concepts (e.g. sequence‐specific DNA binding and TF activity) connected to DEGs in HD were identified using gene set enrichment analysis (GSEA). Finally, in silico drug design was employed to find candidate drugs for the treatment HD, and while the possible modest therapeutic compounds (e.g. cortistatin A, 13,16‐Epoxy‐25‐hydroxy‐17‐cheilanthen‐19,25‐olide, Hecogenin) against HD were expected. Consequently, the results from this study may give researchers useful resources for the experimental validation of Huntington's diagnosis and therapeutic approaches.

## 1 INTRODUCTION

Huntington's disease (HD) is a degenerative brain ailment caused by an increase in CAG (cytosine–adenine–guanine) repeats in the IT15 gene, also known as the huntingtin (HTT) gene.^1^, ^2^ It is transmitted as a dominant characteristic and is fully penetrant. The translation of Huntingtin leads to the creation of a very large polyglutamine domain near the N‐terminus start of the Huntington protein.^3^ Due to the expansion of the CAG region, the mutated huntingtin protein (mHTT) becomes highly unstable. This instability leads to the aggregation of proteins of the same or different kinds and potential disruption of neurotransmission.^4^, ^5^

A complicated interplay between hereditary and environmental variables leads to HD, subsequent generations enduring more dire societal circumstances. Autosomal recessive inheritance often manifests as a diagnosis in middle age, and the disease progresses gradually over 15 to 20 years, with onset occurring between 21 and 50 years of age, averaging 41 years.^6^, ^7^, ^8^ Prevalent signs and indications include loss of fine motor function, anomalies in the cerebellum, gait abnormalities, dysarthria, cognitive problems and stiffness.^9^ It causes significant physical and cognitive impairments, including memory loss, sadness, mood swings, clumsiness and several other psychological difficulties and diseases.^4^, ^10^ When there is strong evidence of a motor condition, namely chorea, together with iatrogenic illnesses and general internal abnormalities, it is considered a clinical diagnosis of HD. While therapies exist for symptomatic relief, there is currently no cure for this debilitating brain disorder.^10^, ^11^ Currently, there are now no drugs available to prevent symptoms and illness progression; nevertheless, there are various successful post‐treatment (i.e. pharmaceutical and nonpharmacologic therapies available).^12^ Furthermore, HD prevalence varies globally, with some locations seeing elevated rates. Genetic testing has led to an increase in the occurrence of the illness in several groups. European and Asian countries have high prevalence rates, whereas South Africa and Zimbabwe have lower percentages.^13^, ^14^, ^15^, ^16^, ^17^ Since 1995, there has been a clear disparity in prevalence rates between Asian nations and white people, with the latter exhibiting greater rates.^16^, ^18^, ^19^

Several studies have suggested histone alterations, protein hubs, transcription factor (TF) difficulties and aberrant microRNA (miRNA) levels as possible indications for diagnosing HD. Diagnosing (HD) in its early stages is difficult due to limits in accuracy, precision and expense.^20^, ^21^ Furthermore, conflicts among researchers have developed about the interpretation of differentially expressed genes (DEGs). Utilising brain cell analysis for early HD identification and therapy sounds promising. The faulty gene responsible with HD was found in 1993, allowing genetic testing for diagnosis.^22^ The genetic test for HD may identify the faulty HTT protein gene, exposing genetic defects in persons without symptoms who are at risk of acquiring the condition later in life.^22^, ^23^ The HTT gene's amplification of CAG triplet repeats cause the manufacture of pathogenic HTT protein residues that are resistant to regular cellular factions, which is the cause of HD.^22^, ^24^, ^25^, ^26^ Most HD patients first suffer motor difficulties, whereas a tiny minority (around 15%) display psychological symptoms before motor abnormalities emerge.^10^, ^27^ The most common manifestation of HD is a specific degeneration of the brain's corpus striatum with no appreciable abnormalities found in the peripheral tissues.^10^, ^28^

Protein hubs, TF deficits, histone changes and aberrant miRNA expression are implicated in HD, and the development of disease‐specific biomarkers is vital for evaluating HD treatments.^29^, ^30^ Various research has focused on discovering molecular biomarkers utilizing brain and blood data. Investigating molecular biomarkers indicating brain expression patterns relevant to HD development is critical.^21^, ^22^, ^24^, ^31^

The objective of this work is to identify molecular biomarkers that indicate the alignment of brain expression patterns with associative factors linked to HD progression. An investigation of DEGs in HD patients was undertaken using data from the GEO database (GSE97100 and GSE74201). An evaluation of probable processes and important genes for HD was carried out, including the over‐representation analysis of KEGG pathways, protein–protein interaction (PPI) network, and Gene Ontology (GO) analysis. Overall, our investigation included cross‐validating the suggested candidate drugs with the latest alternative target proteins utilizing molecular docking as well as molecular dynamics simulation approaches to investigate the probable processes of HD.

## 2 METHOD AND MATERIALS

### 2.1 Data collection

Datasets for the study were obtained from the National Centre for Biotechnology Information (NCBI) Gene Expression Omnibus (GEO) repository.^32^ Homo sapiens data relevant to HD was searched, resulting in 786 datasets. The majority were originally dismissed owing to numerous reasons such as being noncoding, having limited samples, being repetitive, having inadequate formats, improper experimental settings, missing control participants, or coming from non‐human organisms.^19^, ^30^ Ultimately, after careful consideration, two datasets were selected for analysis: GSE97100 containing HDs mRNAseq data and control patient induced pluripotent stem cells‐derived brain microvascular endothelial cells, and GSE74201, which included genomic analysis that highlights disruptions in striatal neuronal development and potential treatment areas in a human neural stem cell model of HD.

### 2.2 Identification of DEG and common gene

**FIGURE 1:**
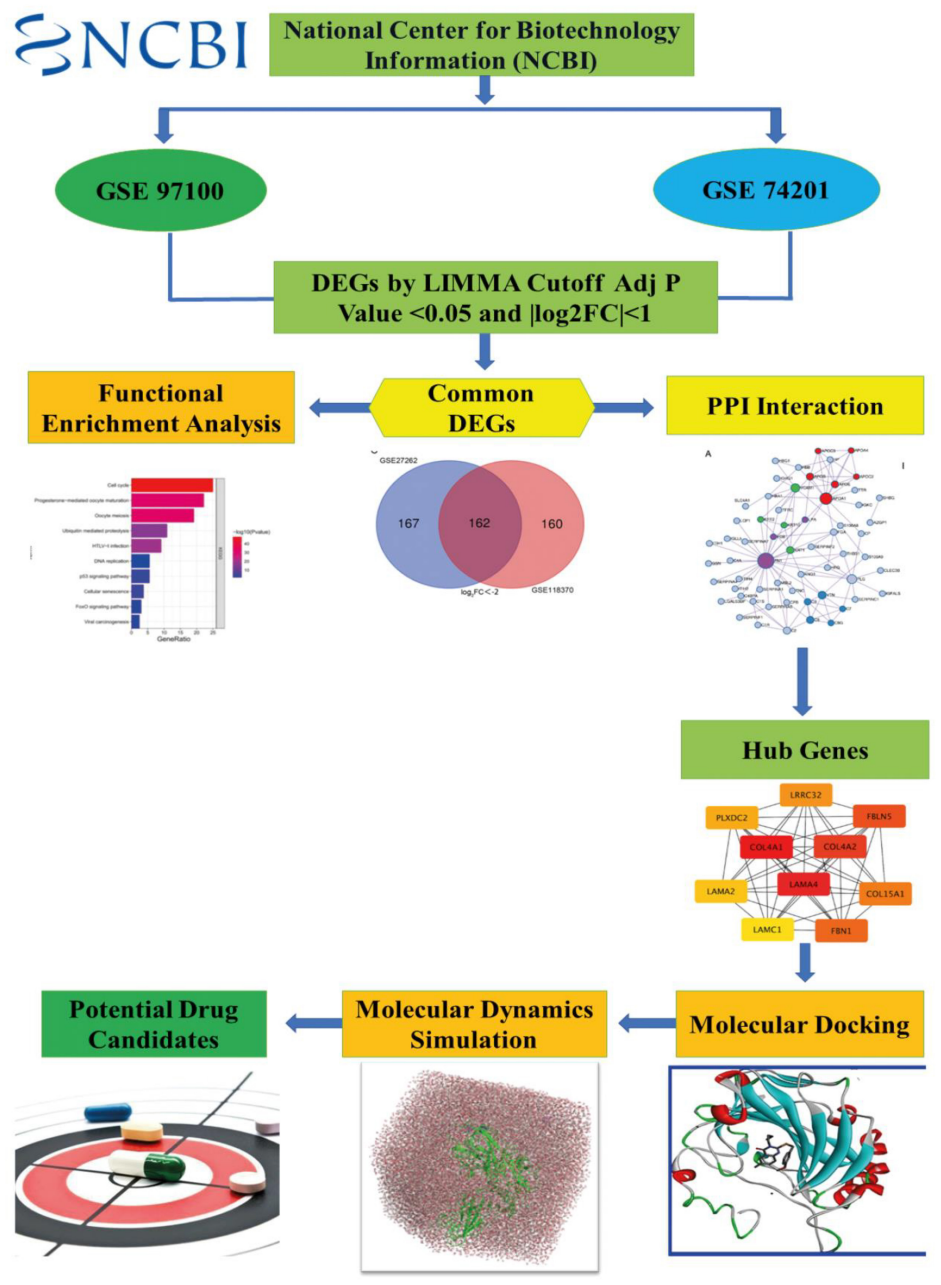
This study identified significant differentially expressed genes (DEGs) associated with HD and analysed related KEGG and Reactome pathways. Using a PPI network and transcriptomic analysis, potential drug targets were identified from the HD expression dataset. Additionally, potential drug candidates targeting these molecules were also discovered.

Gene Set Enrichment Analysis helps in discovering DEGs from a huge gene pool related to illness symptoms by applying various statistical approaches.^19^, ^33^ Figure 1 illustrates the stages engaged in data collecting and notably, GSE97100 and GSE74201 had 46 similar genes, all demonstrating a down‐regulation trend. Following analysis based on characteristics like *p* value <0.05 and the absolute values of log_2_ Fold Control (FC), DEGs were found using linear models using tools like limma from Bioconductor via R, GEO2R and GREIN.^34^, ^35^ *p*‐Value adjustment was conducted employing the Benjamini–Hochberg method, concentrating on false discovery rates. Qe represents the expected value of *Q* ^36^ and it implies that

(i) $$\mathrm{Qe}=E\left(Q\right)$$

The random variable denotes the proportion of errors resulting from null hypotheses that are erroneously rejected, and

(ii) $$Q=V/\left(V+S\right)$$

(iii) R=V+S

where *V* = number of significant true null hypotheses, *S* = number of significant nontrue null hypotheses and *R* = the number of hypotheses that are rejected.

In conclusion, it is apparent that,

(iv) $$E\left(Q\right)=E\left(V/R\right)$$

Overlap analysis of differentially expressed genes (DEGs) was conducted using a Venn diagram to identify common genes.

### 2.3 Enrichment analysis of gene sets

The Enrichr bioinformatics application was used to annotate the KEGG pathway, Reactome pathway and gene ontology of the DEGs.^19^, ^37^ These tools are crucial in understanding gene as well as gene product features across various species, highlighting functional enrichments relevant to biological processes, cellular components and molecular activities.^38^, ^39^, ^40^ The reactome pathway along with the KEGG pathway plays a significant role in comprehending cellular metabolism.^30^, ^41^

### 2.4 Detection of protein–protein interactions

Protein interactions, including protein–protein interaction (PPI), are crucial for biological activities, influencing various biological functions like shape, affinities and permanency of contact.^34^, ^42^, ^43^ PPI networks assisted in identifying protein hub interactions using the STRING database and a specific confidence score of 500 (adjusted *p*‐value less than 0.05).^44^ We utilised Cytoscape software (v.3.10.1)^45^ comprising MCODE and cytoHubba applications^44^ was used to find hub genes.

### 2.5 Identification of transcriptomic regulators

Regulatory molecules like TFs and miRNAs were considered potential regulators affecting DEG expression utilizing Network Analyst.^46^, ^47^ miRNAs hold promise in gene regulation and represent a potential therapeutic target class with developmental roles, impacting diverse physiological activities over time.^19^, ^48^

### 2.6 Protein selection and preparation

Out of the proteins involved in HD formation, we chose one protein that is expressed at unregulated levels for analysis using computer simulations. The proteins of interest in this investigation include P29279‐CCN2_HUMAN (Protein Accession: P29279). The chosen protein P29279‐CCN2_HUMAN (Protein Accession: P29279) was downloaded from UniProt.^49^ The downloaded usual structural format of protein through Uniprot is used straight away for conducting our molecular docking analysis.

### 2.7 Ligand library preparation

A total of 500 marine‐derived compounds were obtained from various literature studies. It contains haliclonacyclamine F, 13,16‐Epoxy‐25‐hydroxy‐17‐cheilanthen‐19,25‐olide, Terreulactone C and many more. The aforementioned structure has been downloaded from PubChem databases including possible structural specifications file format for docking analysis.

### 2.8 Molecular docking

Molecular docking examinations on the previously pointed‐out selected marine‐derived compounds against P29279‐CCN2_HUMAN (Protein Accession: P29279) were performed in auto‐dock Vina using PyRx software, which is openly available and built for molecular docking research investigations. PyRx provides a docking wizard with a simple‐to‐operate user interface that renders it an essential tool for computer‐aided drug design. PyRx further contains a chemical spreadsheet‐like practically and sophisticated display system that is required for rational drug development.^50^, ^51^ The identified pharmacological targets were energy minimized and shifted them into pdbqt format via PyRx.

### 2.9 ADMET

Adsorption, distribution, metabolism and excretion (ADME), as well as drug‐likeness, are vital aspects in assessing the number of potential medicinal compounds that are going to be authorized to advance with clinical trials. Three compounds that displayed the greatest binding energy with P29279‐CCN2_HUMAN (Protein Accession: P29279) were then submitted to the Swiss ADME and pkcsm web server^52^, ^53^ in forecasting their pharmaceutical prognosis and measuring drug similarity, characteristics are evaluated based on Lipinski's Rule of Five.^54^ ADME is a publicly available web‐based application offering robust and swift models for computing drug‐likeness, pharmacokinetic properties and chemistry treatment of phytochemicals.^52^ ProTox‐II web server was applied to measure the lethal dose 50 (LD50) value, cytotoxicity, mutagenicity and neurotoxicity.^55^, ^56^, ^57^

### 2.10 Post‐docking data visualization

The top binding forms, determined by the lowest binding free energy, were selected for three investigated ligands. The interactions between protein and ligands have been further illustrated using Biovia Discovery Studio (https://discover.3ds.com/discovery‐studio‐visualizer‐download)^14^, ^58^ and Ligplot+ (https://www.ebi.ac.uk/thornton‐srv/software/LigPlus/download2.html).^59^ A detailed investigation of every protein–ligand pair was done to seek out hydrogen bonds (H‐bonds), interacting amino acids and particular atoms connected with every ligand.^56^

### 2.11 Molecular dynamics simulation

The stability of the protein‐ligand complex suggesting less dissociation and greater association may be measured using computational approaches. To test the stability of a ligand and its complex with the receptor, a computational molecular dynamics (MD) simulation was done using the Maestro Package inside the Schrödinger platform (paid version) on a Linux OS. The thermodynamic stability was tested utilising the OPLS3e force field with a TIP3P water model.^14^, ^60^ The system was built up with orthorhombic periodic boundary conditions at a distance of 20 Å. Sodium and chlorine ions were introduced for electrical neutralization utilising the OPLS3e force field. MD simulations were done under constant temperature and pressure circumstances (NPT ensemble)^61^ at 300 K and 1 atm utilising Nose‐Hoover temperature coupling and isotropic scaling.^62^, ^63^ A 100 ns simulation was running, storing settings every 100 ps. Stability was examined using statistical methods such as radius of gyration (rGyr), molecular surface area (MoLSA), root mean square fluctuation (RMSF), root mean square deviation (RMSD), solvent accessible surface area (SASA) and intermolecular bond.

## 3 RESULTS

### 3.1 Identifying DEGs

**FIGURE 2:**
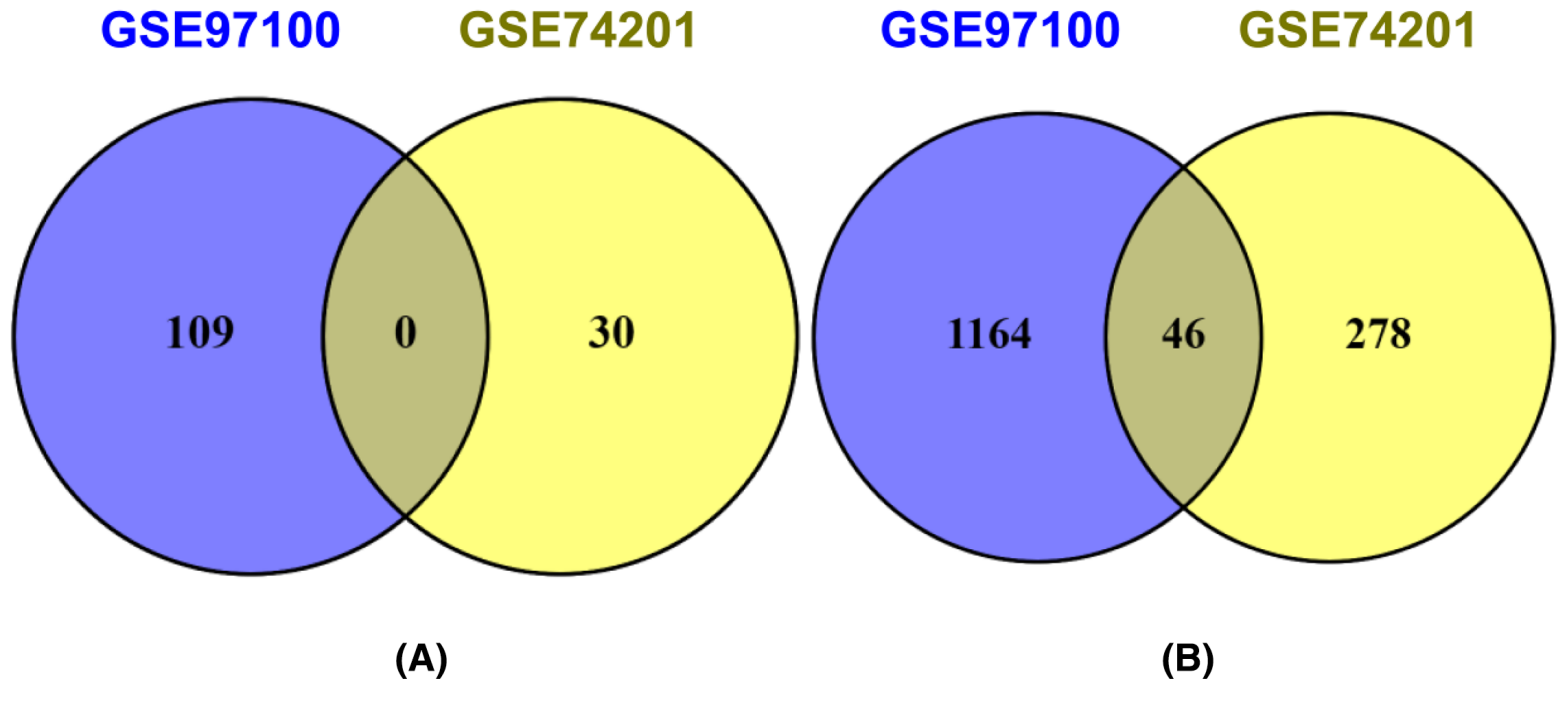
Identification of commonly expressed genes that are upregulated (A) and downregulated (B).

**FIGURE 3:**
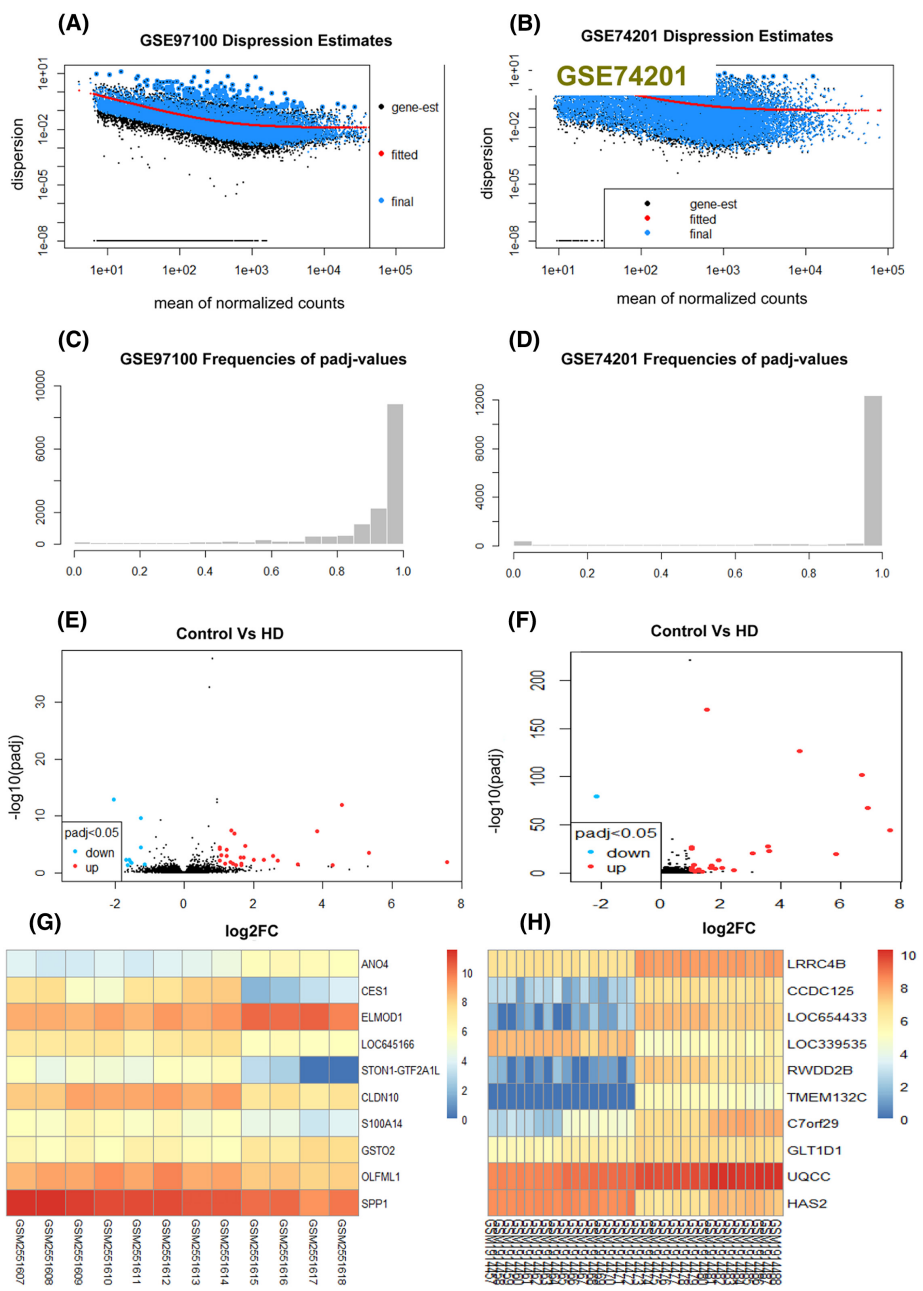
The research identified overlapping differentially expressed genes (DEGs) from various microarray datasets and observed variations in their distribution levels (A and B) Mean Variance Trend Plot (C, D) *p*‐Value Histogram (E, F) Volcano plots indicated significant DEGs (*p* < 0.05, logFC >1 and logFC <−1) compared to inconsequential genes. Genes that are upregulated are represented by red dots, and those that are downregulated by sky‐blue dots. Heatmap for (G, H), illustrating the expression levels of selected datasets, GSE97100 and GSE74201, highlighting differential expression.

Initially, we applied the robust multi‐array average (RMA) approach to normalize the gene expression patterns. Subsequently, we applied the linear models for microarray data (LIMMA) statistical approach to examine the normalized dataset. This analysis revealed 1773 genes that exhibit differential expression (DEGs) between control and the case samples, with significance determined with adjusting *p*‐value of 0.05 and a minimum log_2_ fold change threshold of 1, as depicted in Figure 2. Among these DEGs, 139 were up‐regulated, whereas 1544 were downregulated. Further study was performed based on these DEGs. The transcriptome data for HD identified 46 common DEGs, covering down‐regulated genes from the overlapping DEGs (see Table 1 and Figure 2). Interactive heat maps were created for both the control and case samples using the given datasets presented in Figure 3E,F.

**TABLE 1:** A statistical summary of gene expression in the datasets utilized for analysis.

Serial number	GEO accession	GEO platform	Source	Number Of DEGs			Common genes
				Up	Down	Total	
1.	GSE 97100	GPL16791	iPSC‐derived brain microvascular endothelial cells	109	1210	1319	46
2.	GSE 74201	GPL11154	Human neural stem cell	30	324	354	
TOTAL				139	1544	1773	

### 3.2 Detection of gene ontology and pathway enrichment

**FIGURE 4:**
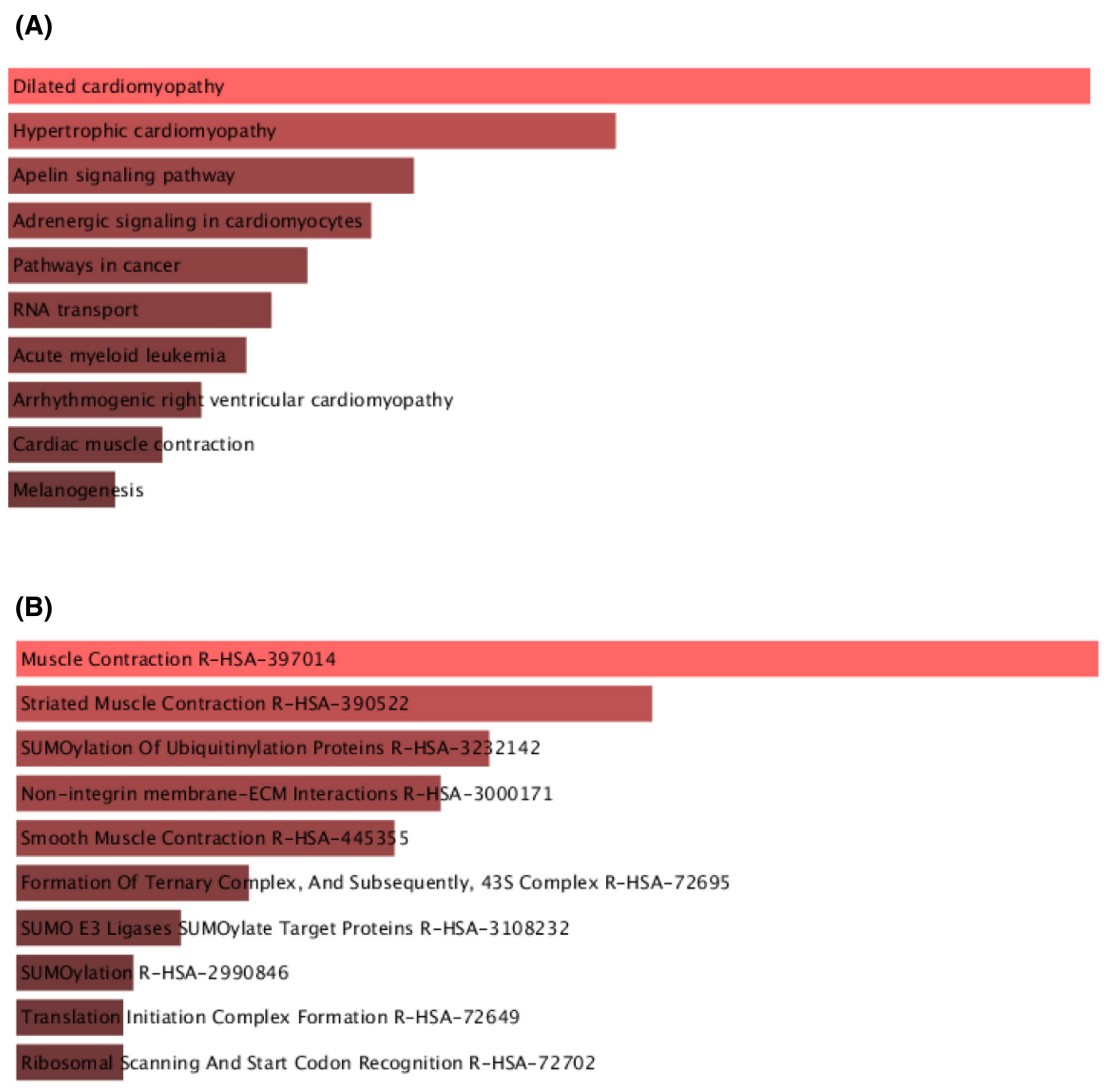
Significantly enriched (A) KEGG pathway, (B) reactome pathway.

Enrichr was utilised to conduct GO term & pathway enrichment analyses, aiming to unveil the biological significance as well as identify enriched pathways linked to our research. GO analysis was applied to understand the biological functions, cellular processes and molecular activities of the DEGs. The top 10 terms within the fields of molecular activities, biological processes and cellular functions are presented in Table 2. Additionally, to determine the biological pathways enriched by the shared DEGs, functional enrichment analysis was carried out. Through the Kyoto Encyclopaedia of Genes and Genomes (KEGG) pathway study, important pathways such as axon guidance, calcium signalling pathway and oxidative phosphorylation were found. In terms of Reactome pathways, enrichment was identified in pathways such as Translation Initiation Complex Formation and SUMOylation Of Ubiquitinylation Proteins. Figure 4A,B exhibit the KEGG pathway and highly enriched reactome pathway, respectively.

**TABLE 2:** Performed gene set enrichment analysis on the genes that exhibited differential expression from microarray data in Huntington's disease (HD) people. Top 10 enriched gene ontology (GO) terms aggregated in a table.

Category	Go ID	Term	Gene Count	*p* (<0.5)	Genes
Biological process	GO:0030336	Negative regulation of cell migration	5	3.54E‐05	DACH1, STK26, TPM1, PHLDB2, ROBO1
	GO:0060048	Cardiac muscle contraction	3	5.92E‐05	TPM1, DMD, SLC8A1
	GO:2000146	Negative regulation of cell motility	4	2.46E‐04	DACH1, STK26, TPM1, ROBO1
	GO:0051492	Regulation Of stress fibre assembly	3	6.85E‐04	TPM1, CCN2, PHLDB2
	GO:0070527	Platelet aggregation	2	0.002933957	CSRP1, ACTN1
	GO:0030239	Myofibril assembly	2	0.00502233	CSRP1, TPM1
	GO:0035771	Interleukin‐4‐mediated signalling pathway	1	0.01144825	STAT5A
	GO:0038043	Interleukin‐5‐mediated signalling pathway	1	0.01144825	STAT5A
	GO:0046885	Regulation Of hormone biosynthetic process	1	0.01144825	STC2
	GO:1904953	Wnt signalling pathway involved in midbrain dopaminergic neuron differentiation	1	0.02051449	WNT3
Cellular component	GO:0030055	Cell‐substrate junction	5	0.002064541	CSRP1, ACTN1, DMD, RND3, PHLDB2
	GO:0042383	Sarcolemma	2	0.006378379	DMD, SLC8A1
	GO:0030175	Filopodium	2	0.008415523	DMD, ACTG2
	GO:0042406	Extrinsic component of endoplasmic reticulum membrane	1	0.01144825	PML
	GO:0005884	Actin filament	2	0.011627547	ACTN1, TPM1
	GO:0005925	Focal adhesion	4	0.01188025	CSRP1, ACTN1, RND3, PHLDB2
	GO:0033179	Proton‐transporting V‐type ATPase, V0 domain	1	0.013722475	ATP6V0E2
	GO:0000220	Vacuolar Proton‐Transporting V‐type ATPase, V0 domain	1	0.013722475	ATP6V0E2
	GO:0098858	Actin‐based cell projection	2	0.01600645	DMD, ACTG2
	GO:0043005	Neuron projection	4	0.038681	FRMD7, PEX6, SLC8A1, ROBO1
Molecular function	GO:0042803	Protein homodimerization activity	8	1.18E‐04	PRPS2, STC2, ACTN1, STK26, DAPK3, TPM1, S100A11, PML
	GO:0030275	LRR domain binding	2	3.37E‐04	DAPK3, ROBO1
	GO:0140035	Ubiquitination‐like modification‐dependent protein binding	2	3.97E‐04	PEX6, PML
	GO:0005524	ATP binding	4	0.00381722	PRPS2, STK26, PEX6, DAPK3
	GO:0003743	Translation initiation factor activity	2	0.004008173	EIF2S3, EIF1AX
	GO:0035639	Purine ribonucleoside triphosphate binding	5	0.004590903	PRPS2, STK26, PEX6, DAPK3, RND3
	GO:0032559	Adenyl ribonucleotide binding	4	0.005474996	PRPS2, STK26, PEX6, DAPK3
	GO:0001665	Alpha‐N‐acetylgalactosaminide alpha‐2,6‐sialyltransferase activity	1	0.01144825	ST6GALNAC5
	GO:0016778	Diphosphotransferase activity	1	0.01144825	PRPS2
	GO:0004571	Mannosyl‐oligosaccharide 1,2‐alpha‐mannosidase activity		0.013722475	MAN1A1

### 3.3 Protein–protein interaction

Figure 6 graphically depicts the protein–protein interaction (PPI) network developed using network analysis, with an emphasis on emphasizing protein hubs. The top 10 hub genes discovered by Cytoscape (v3.10.1) are presented. This graphical depiction demonstrates the relationships among these hub genes, calculated using both Network Analyst and Cytoscape tools (v3.10.1). The PPI network was investigated to discover protein hubs among the DEGs, which comprised 46 common genes. Through this study, hub proteins were found, including TPM1, EIF2S3, CCN2, ACTN1, ACTG2, CCN1, CSRP1, EIF1AX, BEX2 and TCEAL5. These hub genes may act as crucial indications involved in the course of HD.

### 3.4 Transcriptional regulator prediction

To investigate important changes occurring at transcriptional and post‐transcriptional levels, we evaluated DEGs to find TFs and post‐transcriptional regulatory molecules (miRNAs) according to their degree values. Through the finding of TFs and miRNAs targeting DEGs, we hoped to anticipate regulatory chemicals impacting gene expression regulation at these levels. Figure 7A,B exhibit the investigation of TF‐gene and gene‐miRNA coregulatory relationships using network visualization.

Our finding revealed the most significant TFs included CREB1, E2F1, TFAP2A, YY1, RELA, FOXC1, GATA2, NFKB1, HINFP and USF2. Similarly, top important miRNAs discovered were hsa‐mir‐133a‐3p, hsa‐mir‐124‐3p, hsa‐mir‐1‐3p, hsa‐mir‐205‐5p, hsa‐mir‐1277‐5phsa‐mir‐16‐5p, hsa‐mir‐1303, hsa‐mir‐26b‐5p, hsa‐mir‐218‐5p and hsa‐mir‐21‐5p. These regulatory macromolecules are involved in HD and may play key roles in gene regulation at the level of transcription and post‐transcription.

### 3.5 Molecular docking analysis

Molecular docking is an approach that analyses the ligand's optimum binding position with the active region of a target.^64^ Using this approach, the binding region's 3D coordinate space is located in the target, and the binding affinity is computed to determine the orientation of the molecular structure inside the binding site that forms the complex.^64^ The most prominent negative value (either the lowest or greatest binding energy) indicates the most ideal complex configuration created when the ligand binds to the target's active sites, and this value is used to evaluate the importance and sensitivity of binding affinity data.^65^ Furthermore, the molecular docking was carried out to confirm against Huntington for effectiveness of the marine‐derived compounds obtained from the literature study by assessing binding modalities as well as orientations of ligands in the receptor pocket of P29279‐CCN2_HUMAN (Protein Accession: P29279) target. The resulting docking result is displayed in Table 4. It was found that the binding affinity of compounds varied from −9 to −8.6 kcal/mol, which demonstrated their excellent effectiveness. Additionally, the docking outcomes are following the anti‐Huntington actions. Thus, cortistatin A (CID:11561907) phytocompounds were the best inhibitors with the greatest binding affinity of −9 kcal/mol. Therefore, two phytocompounds such as 13,16‐Epoxy‐25‐hydroxy‐17‐cheilanthen‐19,25‐olide (CID:10621161), and Hecogenin (CID:91453) and with their binding affinities of −8.8, −8.6 kcal/mol were reported, respectively (Table 4).

### 3.6 ADMET analysis

Measuring physiological and ADMET characteristics by implementing computational methods is a quick, effective and precise technique.^66^ We explored the physiological and ADMET characteristics of the compounds cortistatin A (CID:11561907), 13,16‐Epoxy‐25‐hydroxy‐17‐cheilanthen‐19,25‐olide (CID:10621161), Hecogenin (CID:91453) by applying SwissADME and pkcsm tool (Table 3). The physiological characteristics, which included the molecular weight of chosen marine‐derived compounds such as cortistatin A (472.62 g/mol), 13,16‐Epoxy‐25‐hydroxy‐17‐cheilanthen‐19,25‐olide (CID:10621161) (402.57 g/mol), Hecogenin (CID:91453) (430.62 g/mol), were within the normally tolerated 150–550 g/mol range of a drug‐like compound. Additional physiochemical parameters, such as quantities of H‐bond acceptors, H‐bond donors, number of rotatable bonds, AMES toxicity and Max. tolerance range of the selected compounds were within the desirable range. It is essential to point out that all compounds displayed drug‐like characteristics and followed the Lipinski rule.^67^ The physicochemical and pharmacological properties of the selected compounds are displayed in Table 3.

**TABLE 3:** Physiochemical and pharmacokinetic properties of three marine‐derived compounds were assessed through ADMET profiling.

Physiochemical									Pharmacological						
Ligands Details	MoW	HAc	HD	NRB	MoR	SA	NLV	DL	IA	BBB	TC	AT	LD50	HT	MDT
Cortistatin A (CID:11561907)	472.62 g/mol	5	2	2	137.45	207.050	0	Yes	High	Yes	0.642	No	2.996	Yes	−0.263
13,16‐Epoxy‐25‐hydroxy‐17‐cheilanthen‐19,25‐olide (CID:10621161)	402.57 g/mol	4	1	1	114.03	174.962	1	Yes	High	Yes	0.316	No	2.058	No	−0.523
Hecogenin (CID:91453)	430.62 g/mol	4	1	0	122.27	187.376	0	Yes	High	Yes	0.315	No	2.028	No	−0.615

Abbreviations: ADMET, absorption, distribution, metabolism, excretion and toxicity; AT, AMES toxicity; BBB, blood–brain barrier; DL, drug‐likeness; HAc, No. of hydrogen bond acceptor; HD, No. of hydrogen bond donor; HT, hepatotoxicity; IA, intestinal absorption; LD50, oral rat acute toxicity, mg/kg; MTD, maximum tolerated dose for a human, log mg/(kg·day); MW, molecular weight; NLV, No. of Lipinski's rule violations; NRB, No. of rotatable bonds; TC, total clearance, log mL/(min·kg).

### 3.7 Post‐docking data visualization

In this work, the interactions between the target protein and a total of three ligands were investigated via Ligplot+ version 2.2 and Biovia Discovery Studio Visualizer 2021. Table 4 and Figure 8 illustrate the results of the docked complex's interactions, which were mostly hydrophobic and hydrogen bonds, as computed through ligplot+ version 2.2. P29279‐CCN2_HUMAN (Protein Accession: P29279) – cortistatin A complex was supported by 10 hydrophobic bonds (Asp334, Ser210, Glu337, Phe336, Lys211, Tyr340, Ala208, Ile217, Ser207 and Ser138), and two hydrogen bonds (Asp140‐2.92 Å, Met139‐2.87 Å) (Table 4 & Figure 8), whereas 13,16‐Epoxy‐25‐hydroxy‐17‐cheilanthen‐19,25‐olide interaction with P29279‐CCN2_HUMAN (Protein Accession: P29279) was supported by eight hydrophobic bonds (Asp334, Ala208, Glu337, Phe336, Ser210, Tyr340, Ile217, Thr219), and three hydrogen bonds Met139‐3.34 Å, Met139‐3.19 Å, Ser207‐2.93 Å. Finally, Hecogenin – P29279‐CCN2_HUMAN (Protein Accession: P29279) demonstrated two hydrogen bonds Cys242‐2.86 Å, Glu243‐2.91 Å and six hydrophobic bonds Met215, Pro241, Lys264, Ile266, Met238 and Glu269.

**TABLE 4:** List of compound identity, the chemical name of selected best three ligands and the binding affinity of ligands with P29279‐CCN2_HUMAN (Protein Accession: P29279) receptor and the thorough intermolecular interactions between them.

S.N.	Compound CID	Chemical formula	Binding score (Kcal/mol)	Amino acid interaction	
				Hydrogen bond interaction	Hydrophobic bond interaction
01	CID:11561907	Cortistatin A	−9	Asp140‐2.92 Å, Met139‐2.87 Å	Asp334, Ser210, Glu337, Phe336, Lys211, Tyr340, Ala208, Ile217, Ser207 and Ser138
02	CID:10621161	13,16‐Epoxy‐25‐hydroxy‐17‐cheilanthen‐19,25‐olide	−8.8	Met139‐3.34 Å, Met139‐3.19 Å, Ser207‐2.93 Å	Asp334, Ala208, Glu337, Phe336, Ser210, Tyr340, Ile217 and Thr219
03	CID:91453	Hecogenin	−8.6	Cys242‐2.86 Å, Glu243‐2.91 Å	Met215, Pro241, Lys264, Ile266, Met238 and Glu269

### 3.8 MDS analysis

Molecular dynamics simulation (MDS) assessing the structural stability of atoms and molecules involves representing their systems at the atomic level. Also, molecular dynamics (MD) simulation stands out as a unique method for evaluating the stability of a ligand in association with a particular protein macromolecule. In this instance, a molecular dynamics (MD) simulation lasting 100 nanoseconds was conducted to determine the stability of the protein‐ligand complex. The following was carried out to evaluate the ability of ligands to efficiently bind to the protein, particularly targeting its active site region. The results of the molecular dynamics (MD) simulation have been documented, relying on SASA, protein‐ligand contact analysis (P‐L contact), MoLSA, RMSF, RMSD and intramolecular hydrogen bonds (Intra HB).

#### 3.8.1 Root mean square deviation (RMSD) analysis

The root mean square deviation (RMSD) of a protein‐ligand complex system calculates the average distance that a particular atom moves from its original position over a defined period. Typically, it involves taking the square root of the average of squared errors to calculate the amount of variation between two values, namely the observed and estimated values. The average, or mean value that variations across frames within the range of 1–5 Å or 0.1–0.5 nm are acceptable, but a value surpassing this range suggests a significant conformational change in the protein.^57^ The combined RMSD analysis of the complex structures involving the drug candidates CID‐11561907 (depicted in green), CID‐10621161 (in yellow) and CID‐91453 (in orange) with the protein P29279‐CCN2_HUMAN (Protein Accession: P29279) was conducted to observe alterations in their order, as illustrated in Figure 9. The RMSD values for the three compounds fell within the range of 3.6 Å to 4.8 Å, showing slight fluctuations between 4 and 28 ns that were entirely within acceptable limits.

#### 3.8.2 Root mean square fluctuation (RMSF) analysis

The root mean square fluctuation (RMSF) may aid in detecting and determining the regional modifications that occur within the protein chain whenever a specific ligand interacts with certain protein residues.^57^ Consequently, the RMSF values for compounds CID: 11561907, CID:10621161 and CID:91453 while in combination with P29279‐CCN2_HUMAN (Protein Accession: P29279) were computed. This aimed to discern alterations in protein flexibility due to the binding of different ligand molecules to a specific protein residue site, as illustrated in Figure 10. When RMSF's change value is greater than 5 Å, it is considered a tangible and significant change that occurs in amino acid residue‐specific flexibility. The RMSF graph indicated average low and significant values of the P29279‐CCN2_HUMAN (Protein Accession: P29279)‐CID: CID:91453 (Orange) (4–5 Å), P29279‐CCN2_HUMAN (Protein Accession: P29279)‐CID:10621161 (Yellow) (3.3–4.5 Å) and P29279‐CCN2_HUMAN (Protein Accession: P29279)‐CID: 11561907 (Green) (3.1–4.8 Å), showing slight fluctuations between 5 and 50 and 170 and 200 residue index, indicating that the natural compounds were strongly bound to P29279‐CCN2_HUMAN (Protein Accession: P29279) protein in terms of their average positions and shown in Figure 10.

#### 3.8.3 Radius of gyration (Rg) analysis

The distribution of atoms around the axis of the protein‐ligand complex is known as its radius of gyration. Rg measurement has become among the most significant indicators for macromolecule structural stability because it reflects modifications to complex compactness. Therefore, in the study of Rg analysis, the compactness of selected compounds CID:11561907, CID:10621161 and CID:91453 in complex with P29279‐CCN2_HUMAN (Protein Accession: P29279) protein was also studied during 100 ns simulation time in Figure 11.

#### 3.8.4 Solvent accessible surface area

Solvent‐accessible surface area (SASA) is a method used to assess the polar and non‐polar surface area of molecules, helping to understand how residues interact with the solvent.^68^ According to the findings shown in Figure 12, the SASA values for the CID:11561907‐P29279‐CCN2_HUMAN (Protein Accession: P29279) complex, CID:10621161‐P29279‐CCN2_HUMAN (Protein Accession: P29279) and the CID:91453‐P29279‐CCN2_HUMAN (Protein Accession: P29279) complex are determined to be 500.49 A,^2^ 120.038 A,^2^ and 140.148 A,^2^ respectively. It is worth mentioning that the CID:10621161‐P29279‐CCN2_HUMAN (Protein Accession: P29279) complex exhibits a decreased SASA in comparison to both the CID:91453‐P29279‐CCN2_HUMAN (Protein Accession: P29279) and CID:11561907‐P29279‐CCN2_HUMAN (Protein Accession: P29279) complex, suggesting that CID:10621161 induces conformational changes.

#### 3.8.5 MoLSA analysis

The molecular surface area (MoLSA) of the ligands exhibited remarkable variations among the simulations. All phytocompounds, CID:10621161 (Yellow), CID:91453 (Orange) and CID:11561907 (Green) consistently displayed the lowest MoLSA when coupled to P29279‐CCN2_HUMAN (Protein Accession: P29279), ranging between 0 and 5 Å^2^ (Figure 13).

#### 3.8.6 Intramolecular bond

The protein–ligand interaction, notably involving hydrogen bonds, hydrophobic interactions and water bridges, greatly impacts drug selectivity, metabolism and absorption. Hence, the intermolecular interactions of the protein–ligand complexes were investigated using simulation interaction diagrams (SIDs) throughout a 100 ns simulation period. The interaction fraction values were determined for the protein and ligands, including compounds CID:10621161, CID:91453 and CID:11561907 and presented in Figure 14. Among these compounds, CID:91453, CID:10621161 and CID:11561907 demonstrated the greatest interaction fraction values of 0.9, 0.65 and 0.9, respectively, at amino acid residues GLU 269, GLU 327 and ASP 37. These interactions featured numerous bonds, suggesting strong binding. Compounds CID:10621161 and CID:91453 demonstrated the greatest water bridges and hydrogen bonds, indicating improved compound stability.

## 4 DISCUSSION

Huntington's disease an inherited neurologic illness often arises in adults in their forties and fifties.^25^, ^69^ Recent research has connected the dysregulation of possible biomarkers to neurological and neurodegenerative illnesses,^70^, ^71^ with multiple investigations addressing the influence of biomolecular activities on HD patients.^31^, ^72^, ^73^, ^74^, ^75^ Currently, omics‐based methodologies are widely applied in biomedical and systems biology investigations, showing to be important tools in dissecting disease pathophysiology, uncovering molecular pathways and generating biomarkers for diverse illnesses.^34^ Previous discoveries show that gene expression may be controlled during various phases of RNA processing, protein post‐translational modifications (PTMs), translation, or genetic modifications.^76^, ^77^ Characterizing target protein activities in bioactive compounds is critical for defining the biochemical route of a certain illness and understanding the participation of basic processes in a particular phenotype.^34^, ^78^ Investigation into hub proteins has received interest, with protein–protein interactions (PPI) defined as either persistent or transient depending on their length and function.^30^, ^79^ Networks built using PPI are considered scale‐free, with component connections usually following a Poisson distribution.^80^ Integrating a network‐based method with genomic data assists in detecting relationships between diverse biological processes and activities, leading to the identification of novel pathways, interaction networks and disease‐related signals that help establish biomarkers and therapeutic targets.^81^ While previous studies have examined miRNA expression levels in cellular as well as mouse models,^75^ shared gene expression patterns in individuals with HD,^82^ and DNA methylation in HD.^72^ There is a lack of comprehensive bioinformatics analysis that investigates molecular markers and pathways in both healthy persons and those with HD. To overcome this gap, a complete bioinformatics strategy was utilised to disclose molecular markers and essential pathways for HD in this work, offering an encompassing view.

Using the term “HD,” we ran a search in the GEO database, extracting records containing mRNA expression profiles of *Homo sapiens*. After extensive study of the available literature, these datasets were separated into control and illness groups.^31^ Employing a bioinformatics method, we evaluated DEGs across these groups, indicating substantial differences in gene expression among HD patients compared to neurologically healthy controls. Two datasets, GSE97100 and GSE74201, were selected, leading to the identification of 46 commonly down‐regulated genes through systematic and statistical approaches (Figure 2).

Gene ontology (GO) analysis was then undertaken to study the biological implications of these 46 DEGs linked with HD. The identified genes were observed to encode proteins with diverse molecular functions, associated with various fundamental biological processes such as Protein Homodimerization Activity, alpha‐N‐acetylgalactosaminide Alpha‐2,6‐Sialyltransferase Activity, Ubiquitination‐Like Modification‐Dependent Protein Binding, ATP Binding, Purine Ribonucleoside Triphosphate Binding, Adenyl Ribonucleotide Binding, Translation Initiation Factor Activity, Diphosphotransferase Activity, LRR Domain Binding and Mannosyl‐Oligosaccharide 1,2‐Alpha‐Mannosidase Activity. Several other relevant studies have also identified GO terms related to signal transduction in the Biological Process^73^, ^83^ and plasma membrane in the Cellular Component.^74^ In our analysis, we observed enrichment of molecular function related to TF activity and sequence‐specific DNA binding by gene ontologies. Additionally, a previous study demonstrated the relevance of TF activity in molecular function^3^, ^84^ to HD. By merging historical data with our results, new treatment targets or putative pathogenic pathways for future investigation may be identified.

**FIGURE 5:**
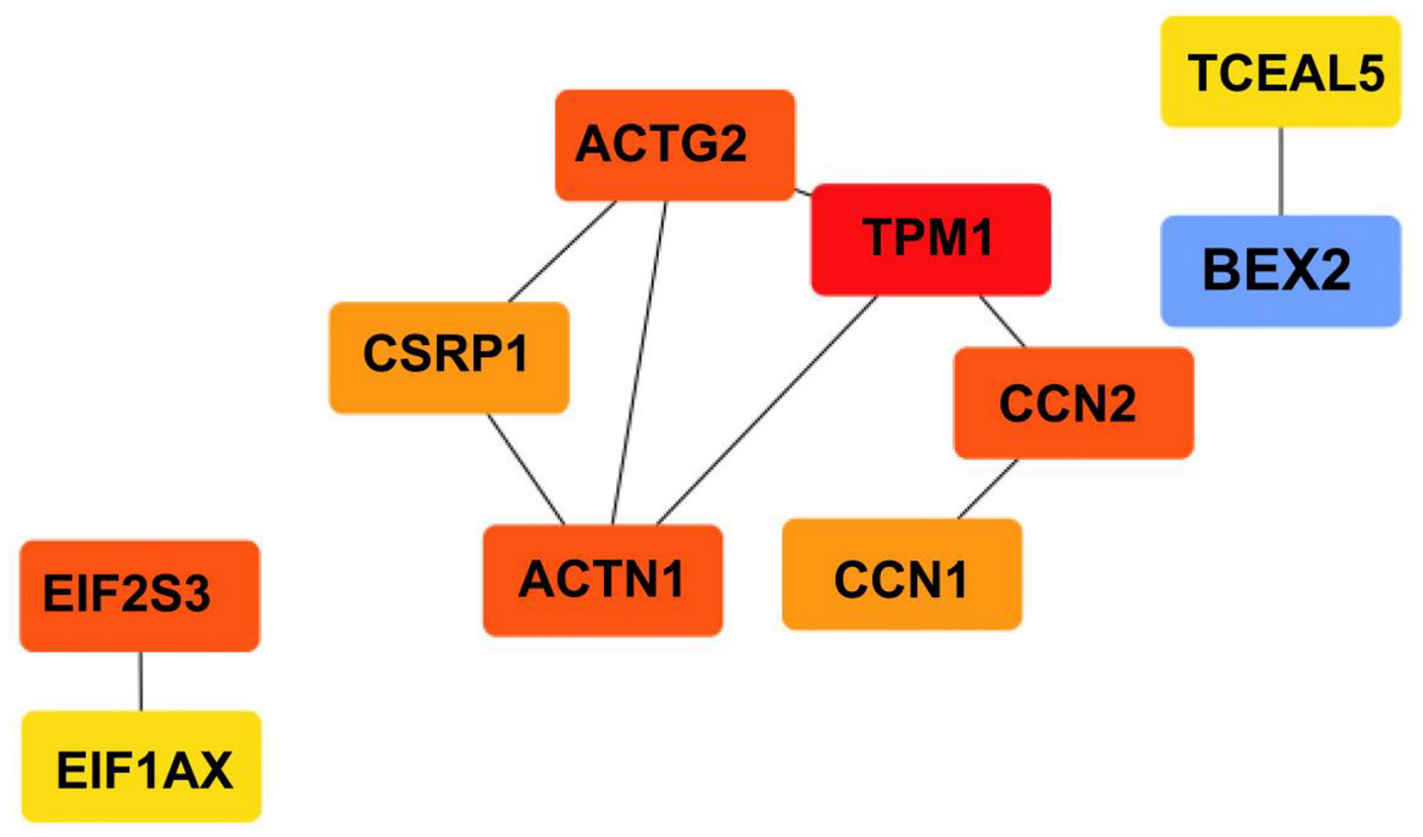
Top 10 Hub genes identified by Cytoscape.

**FIGURE 6:**
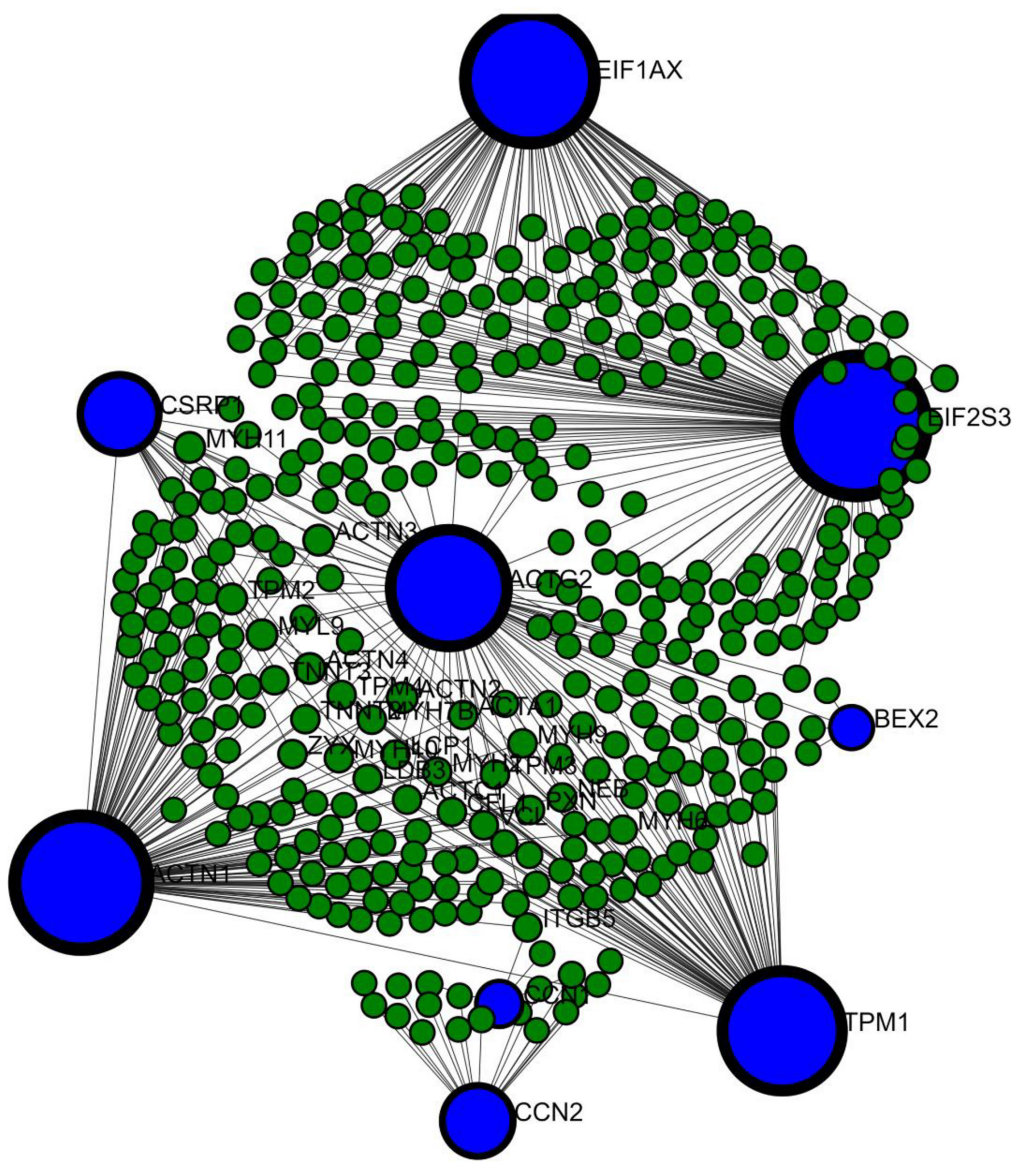
The Network Analyst server was applied to visualize and forecast hub proteins within the Protein–Protein Interaction (PPI) Network. A protein–protein interaction (PPI) network was developed, emphasizing the top 10 hub genes and their interactions with additional Differentially Expressed Genes (DEGs). A confidence score of 500 was utilized in the construction of this network using the STRING interactome database. The visual representation depicts the top 10 hub genes as blue nodes, DEGs as green nodes and the degree of interactions among DEGs as blue edges. Larger nodes indicate hub proteins, while smaller nodes represent DEGs.

Moreover, *TPM1, EIF2S3, CCN2, ACTN1, ACTG2, CCN1, CSRP1, EIF1AX, BEX2* and *TCEAL5* were identified as important hub genes, highlighting their potential as biomarkers for HD (Figures 5 and 6). These hub proteins are considered to have essential roles in the pathways causing the illness.^75^ Subsequently, a protein interaction network concentrating on DEGs was rebuilt to reveal important hub proteins contributing to the genesis and progression of HD. For example, TPM1 encodes Tropomyosins, a well‐conserved group of actin‐binding proteins vital in different physiological processes.^85^ Humans contain four tropomyosin genes that undergo alternative splicing, yielding different isoforms critical for muscle function and involved in many muscle‐related diseases.^86^

The EIF2S3 gene encodes the γ subunit of the eIF2 complex, crucial for beginning protein synthesis and regulating stress response.^87^ Mutations in EIF2S3 have been associated with severe neurological diseases including MEHMO syndrome.^88^, ^89^, ^90^, ^91^ Additionally, CTGF/CCN2 is a matricellular protein of the CCN family, involved in different cellular processes such as cell proliferation, motility and ECM synthesis. Its fibrotic action has been widely investigated, notably in illnesses involving fibrosis such as Duchenne muscular dystrophy.^92^, ^93^ Therefore, Alpha actinin 1 helps tie the myofibrillar actin filaments to the Z‐line and it's a crucial player in muscle contraction. The sarcomeric Z‐line acts by joining “titin and actin filaments from opposing sarcomere halves in a lattice connected by alpha‐actinin.”^94^ Mammals, such as humans, have four α‐actinin encoding genes (ACTN1, ACTN2, ACTN3 and ACTN4). ACTN1 was researched for the aim of this experiment. Actinins are crucial for muscular contraction, and disruption of their normal function may lead to muscle conditions such as hereditary inclusion body myopathy.^95^ Previous investigations have shown alternatively spliced mRNAs of ACTN1.^96^, ^97^

There are two alternatively spliced isoforms; for this research, they include Titin‐L and Titin‐S. In addition, CCN1 has shown neuroprotective benefits in several neurodegenerative disorders. For instance, Chen et al.^98^ revealed that CCN1 protected against ischemia‐induced neuronal damage in rats. Although this work did not explicitly address HD, it shows that CCN1 may have neuroprotective qualities that might be relevant to HD, where neurons are prone to injury and degeneration.^99^, ^100^ BEX2 has been involved in enhancing neuronal differentiation and neurite outgrowth. In neurodegenerative illnesses such as HD, preserving neuronal integrity and encouraging neural plasticity are critical for minimising disease progression. BEX2's involvement in neuronal development might alter the survival and function of neurons damaged by HD disease.^101^, ^102^ TCEAL5 has been linked to cellular differentiation processes. In neurodegenerative illnesses like HD, maintaining appropriate neuronal development and function is critical for reducing disease progression. Although there may not be direct evidence connecting TCEAL5 to HD, its involvement in cellular differentiation might alter neuronal integrity and function in the setting of HD.^103^, ^104^

**FIGURE 7:**
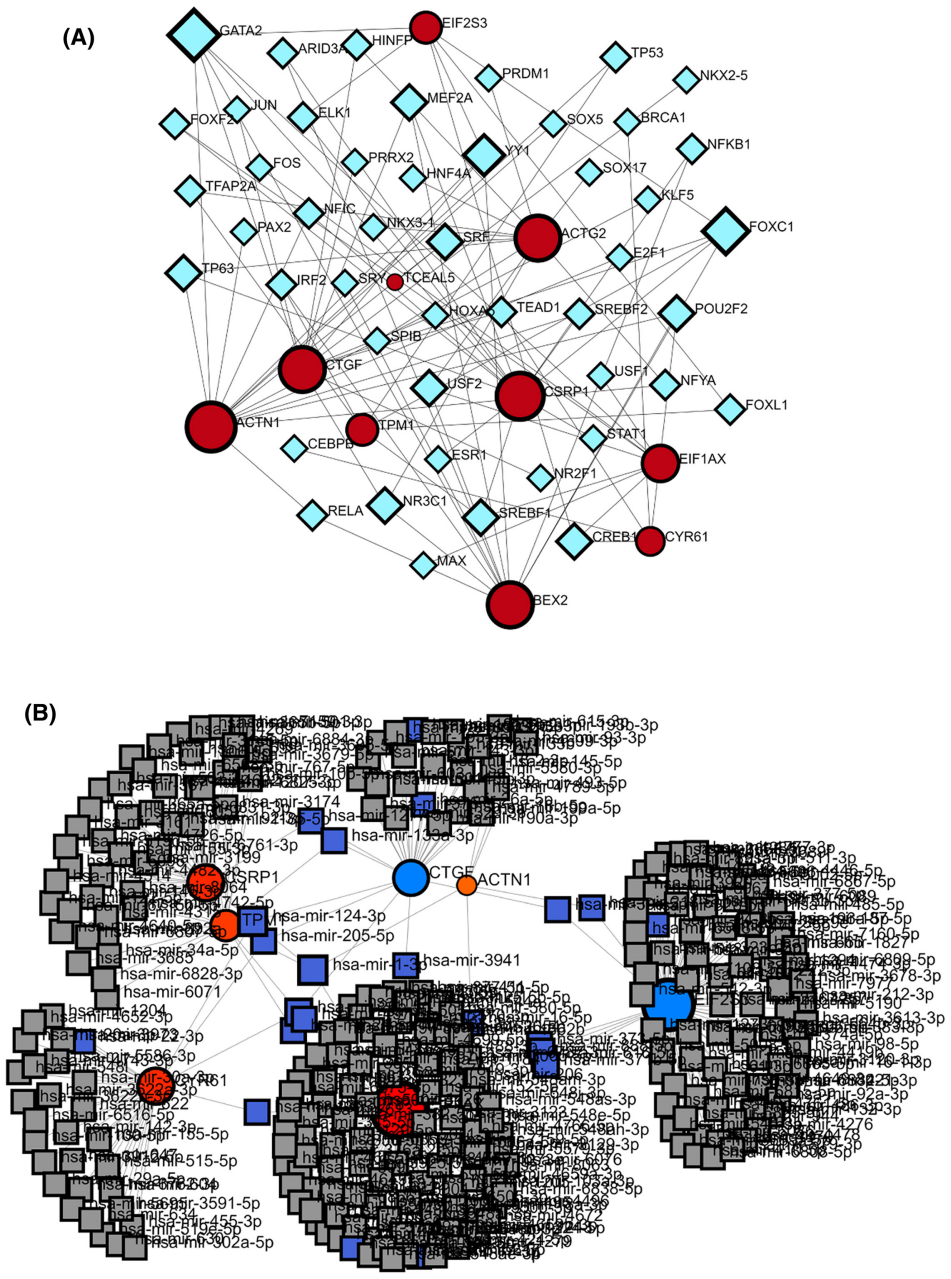
The molecules identified as the transcriptomic signature from the Network Analyst server are visualized in two parts:(A) The top 10 transcription factors (TFs) linked to differentiated expressed Genes (DEGs) are displayed within the network. In this visualization, red nodes signify TFs, ash‐coloured nodes represent DEGs and the edges indicate interactions among DEGs (B) The top 10 microRNAs (miRNAs) associated with DEGs are showcased. The diagram displays brown square nodes for miRNAs and red nodes for DEGs. The connections between the nodes signify interactions between the DEGs.

The exploration of directory biomolecules as possible biomarkers for major diseases like neurodegenerative disorders is increasing.^29^, ^34^, ^105^, ^106^ We examined how TFs and miRNAs are involved in regulating DEGs via TF‐DEG.^73^, ^82^ MicroRNAs play a vital function in gene expression control and show potential as biomarkers for HD and other illnesses. Several miRNAs are anticipated to have a role in the pathogenesis of HD.^2^, ^107^ Our research unveiled the most noteworthy transcriptomic factors (TFs), that is, GATA2, E2F1, HINFP, TFAP2A, RELA, CREB1, FOXC1, NFKB1, YY1 and USF2 were identified as significant TFs, while hsa‐mir‐1‐3p, has‐mir‐1303, hsa‐mir‐26b‐5p, hsa‐mir‐1277‐5p, hsa‐mir‐133a‐3p, hsa‐mir‐16‐5p, hsa‐mir‐205‐5p, hsa‐mir‐21‐5p, hsa‐mir‐218‐5p and hsa‐mir‐124‐3p were discovered as top miRNAs implicated in HD (Figure 7). FOXC1, GATA2 and YY1 were found as regulatory TFs in several neurological conditions, such as Alzheimer's disease and various others.^71^, ^105^ In a research investigation, scientists observed an elevation in TFAP2A nucleoid signal concentration within specific micropattern colonies associated with HD.^108^

Downregulation of hsa‐miR‐124‐3p is involved in various neurological disorders such as Alzheimer's disease and HD in mice and humans.^109^ Thus, abnormal expression of miR‐124 has been detected in HD, ischemic stroke, Alzheimer's disease, Parkinson's disease and hypoxic–ischemic encephalopathy.^109^, ^110^ Besides, other miRNAs include hsa‐miR‐26b‐5p, which is connected with neuronal differentiation, development and vitamin D metabolism.^111^, ^112^

In recent times, *in‐silico* drug design has emerged as a crucial and essential technology in modern drug development, offering the potential to significantly decrease the cost, time and labour associated with the drug discovery process.^113^, ^114^ By enabling scientists to target their biological and synthetic research efforts more precisely than was previously feasible, it has aided in the development of novel medications by lowering costs and shortening research timeframes.^115^, ^116^

**FIGURE 8:**
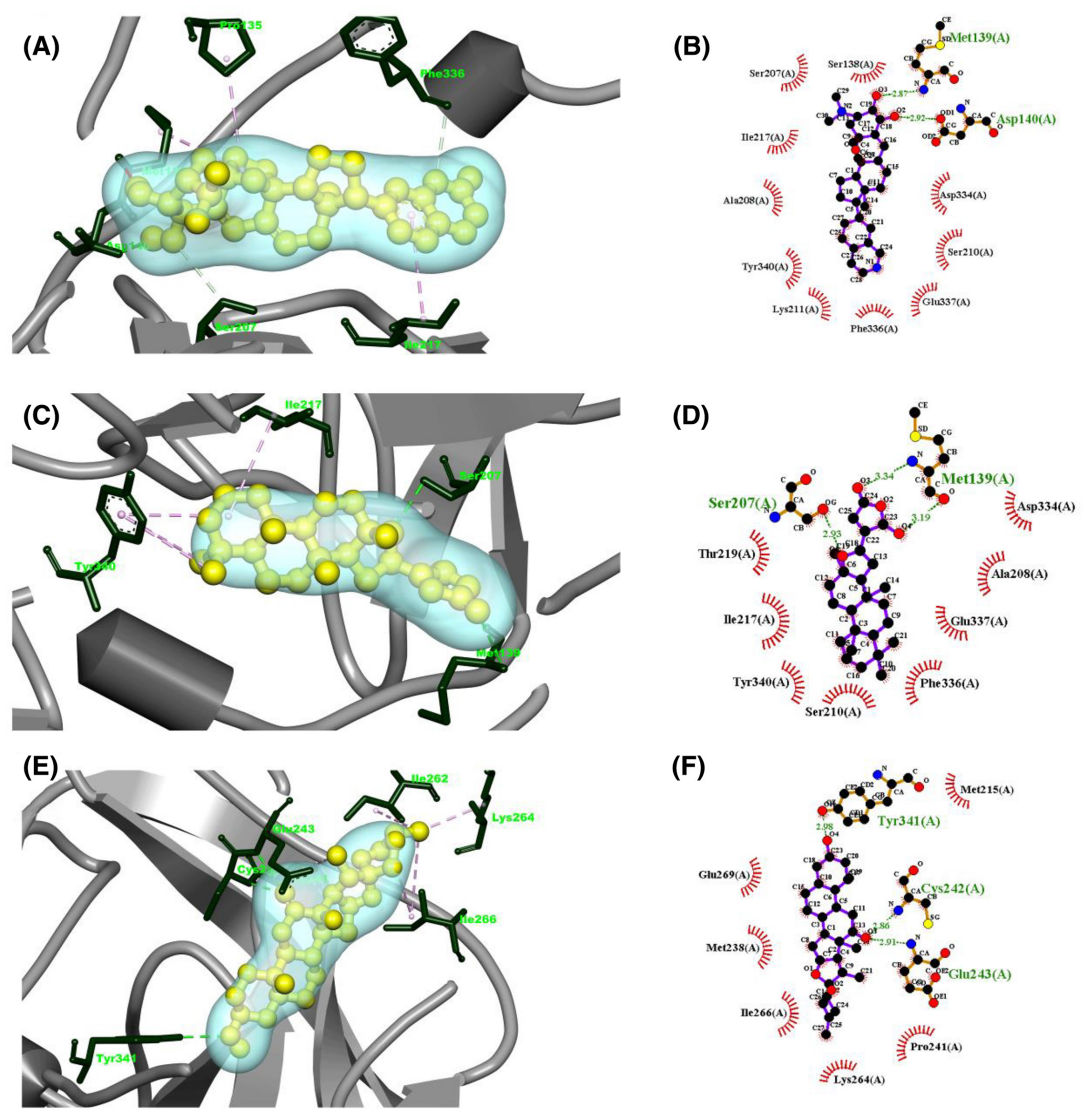
The depiction shows the interaction between selected phytocompounds and P29279‐CCN2_HUMAN (Protein Accession: P29279). On one side, we have the three‐dimensional complex of protein–ligand interaction, and on the other, we have the two‐dimensional complex. (A, B) cortistatin A—P29279‐CCN2_HUMAN (C, D) 13,16‐Epoxy‐25‐hydroxy‐17‐cheilanthen‐19,25‐olide—P29279‐CCN2_HUMAN (E, F) Hecogenin‐P29279‐CCN2_HUMAN.

Nowadays, a lot of researchers are using *in‐silico* drug design techniques since they can speed up the process of creating high‐quality drugs. This is accomplished by Molecular Dynamic Simulation (MDS), post‐docking interaction analysis, molecular docking result assessment and computer‐aided drug discovery of potential therapeutic compounds for various diseases.^113^, ^114^ Molecular docking is a method used to forecast how molecules may interact optimally in terms of their structure and with the least potential binding strength. The molecular docking method was initially used to choose medications based on which ones had the lowest binding affinities. The notable docking outcome indicates that three compounds out of 500 marine‐derived compounds could be considered for further exploration as potential therapeutic candidates against HD by targeting protein and gene expression pathways. Three marine‐derived compounds, namely cortistatin A, 13,16‐Epoxy‐25‐hydroxy‐17‐cheilanthen‐19,25‐olide and hecogenin, exhibited the most favourable docking scores of −9 kcal/mol, −8.8 kcal/mol and −8.6 kcal/mol, respectively, when interacting with the protein P29279‐CCN2_HUMAN (Protein Accession: P29279) and exhibit considerable inhibitor against HD. Analysing the binding interaction, strong hydrophobic and hydrogen interactions were found between the ligands and the protein (Figure 8). Furthermore, molecular dynamics simulations can be employed to validate the stability of a protein within a compound that includes ligands.^117^, ^118^ Additionally, it can assess the flexibility and stability of complexes formed between proteins and ligands within a defined simulated environment, such as the human body.

**FIGURE 9:**
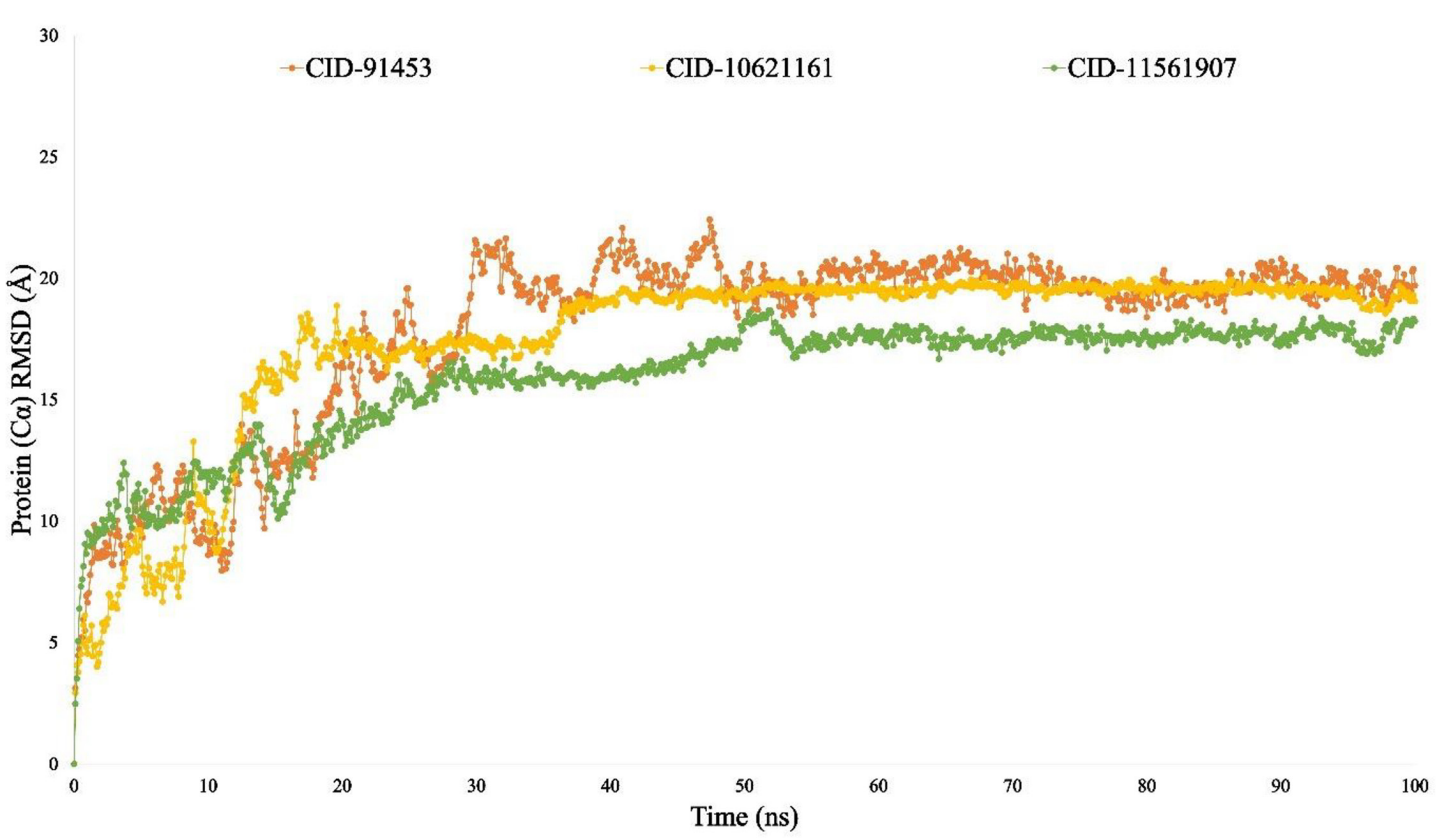
RMSD values of the complex structure derived from Cα atoms are shown in the line graph, viz: CID‐11561907 (depicted in green), CID‐10621161 (in yellow) and CID‐91453 (in orange).

**FIGURE 10:**
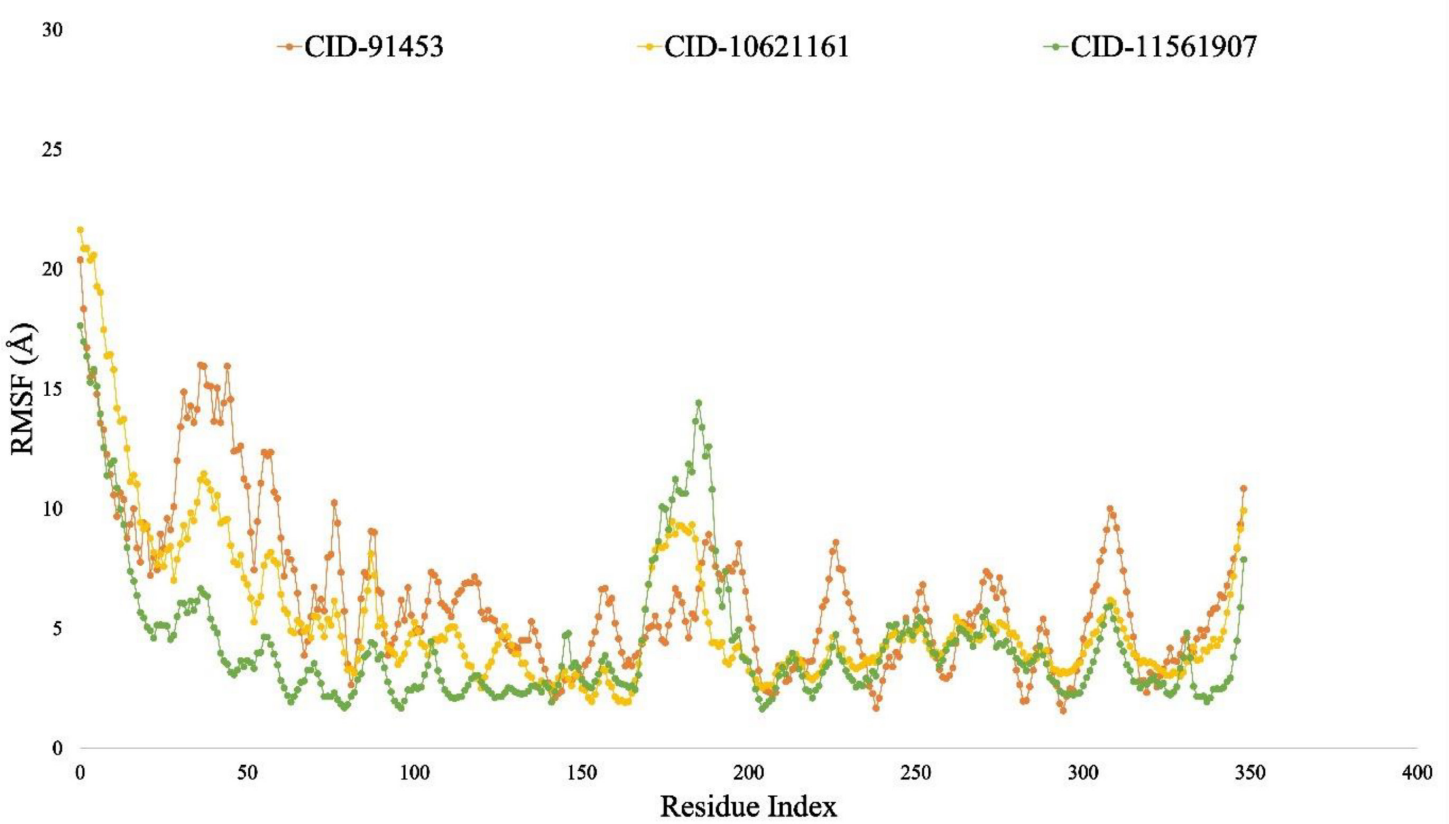
Showing the RMSF values taken from protein residues Cα atoms of the complex structure, viz CID‐11561907 (depicted in green), CID‐10621161 (in yellow) and CID‐91453 (in orange).

**FIGURE 11:**
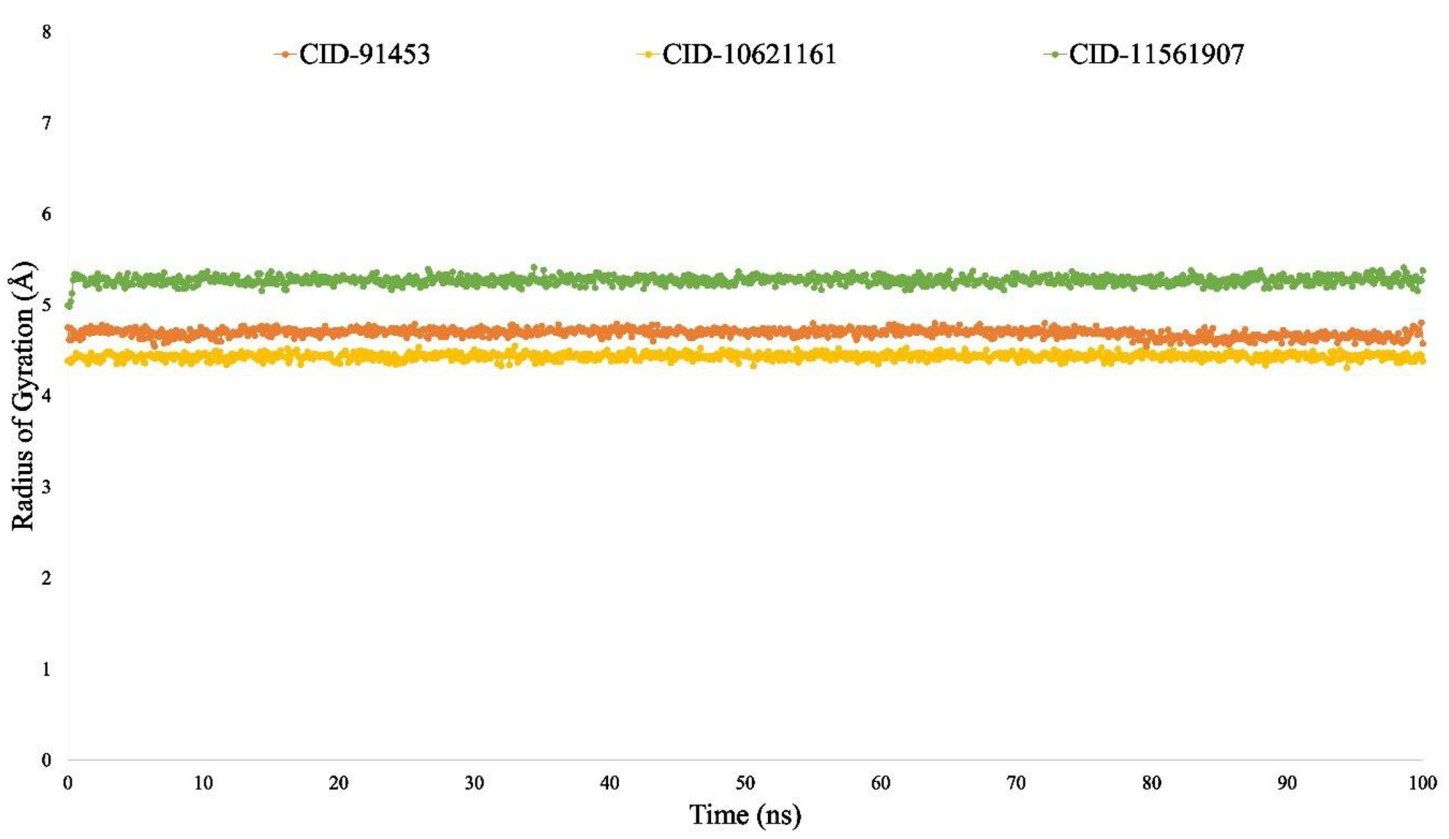
The analysed radius of gyration results of selected three compounds CID:91453, CID: 10621161, CID: 11561907 with P29279‐CCN2_HUMAN (Protein Accession: P29279) protein are displayed by orange, yellow and green, respectively.

**FIGURE 12:**
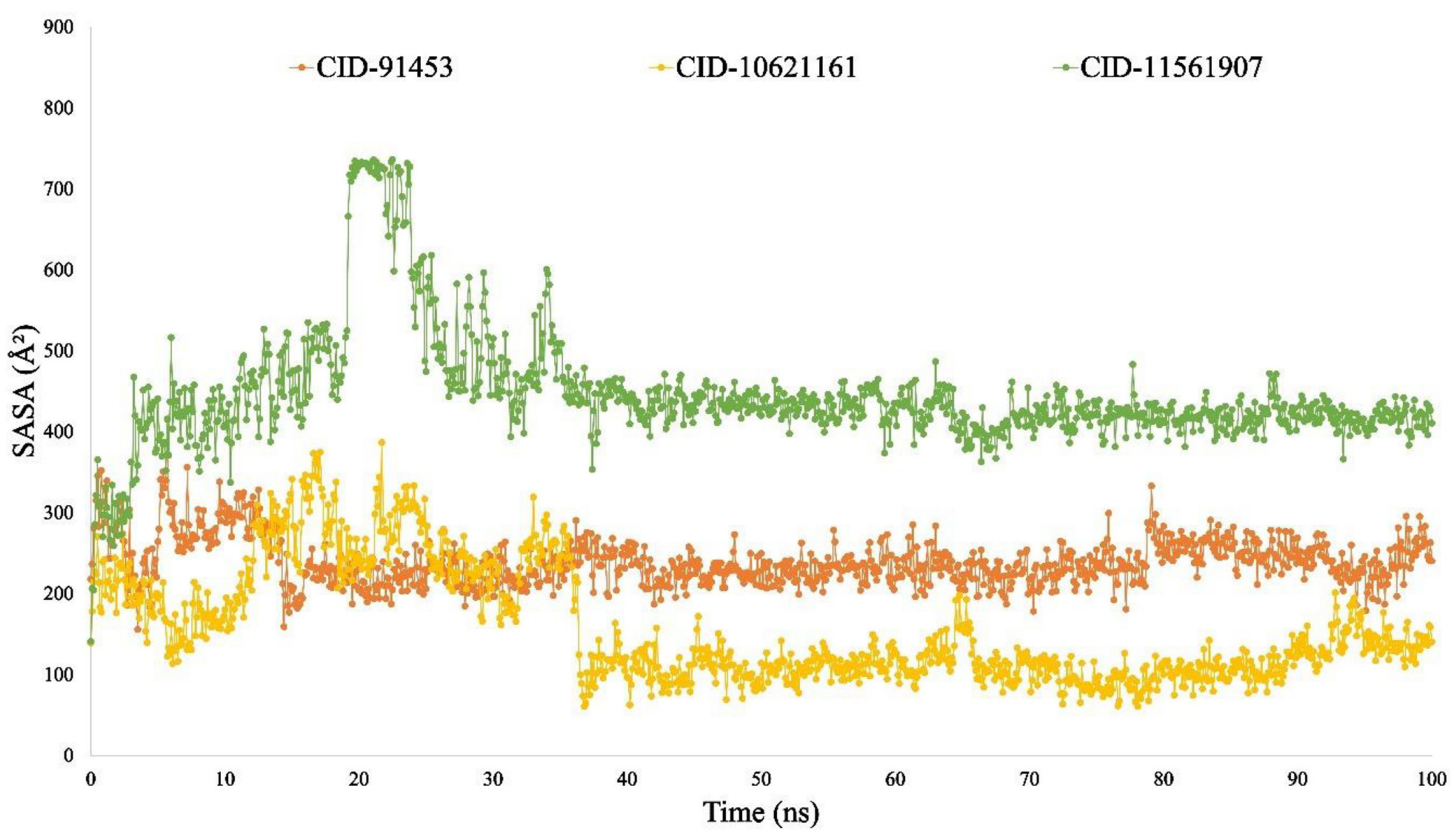
The 100 ns simulation diagram was used to determine the solvent‐accessible surface area (SASA) of the protein‐ligand interaction. The selected three ligands' compounds CID:91453 (Orange), CID: 10621161 (Yellow) and CID: 11561907 (Green) in association with the selected protein.

**FIGURE 13:**
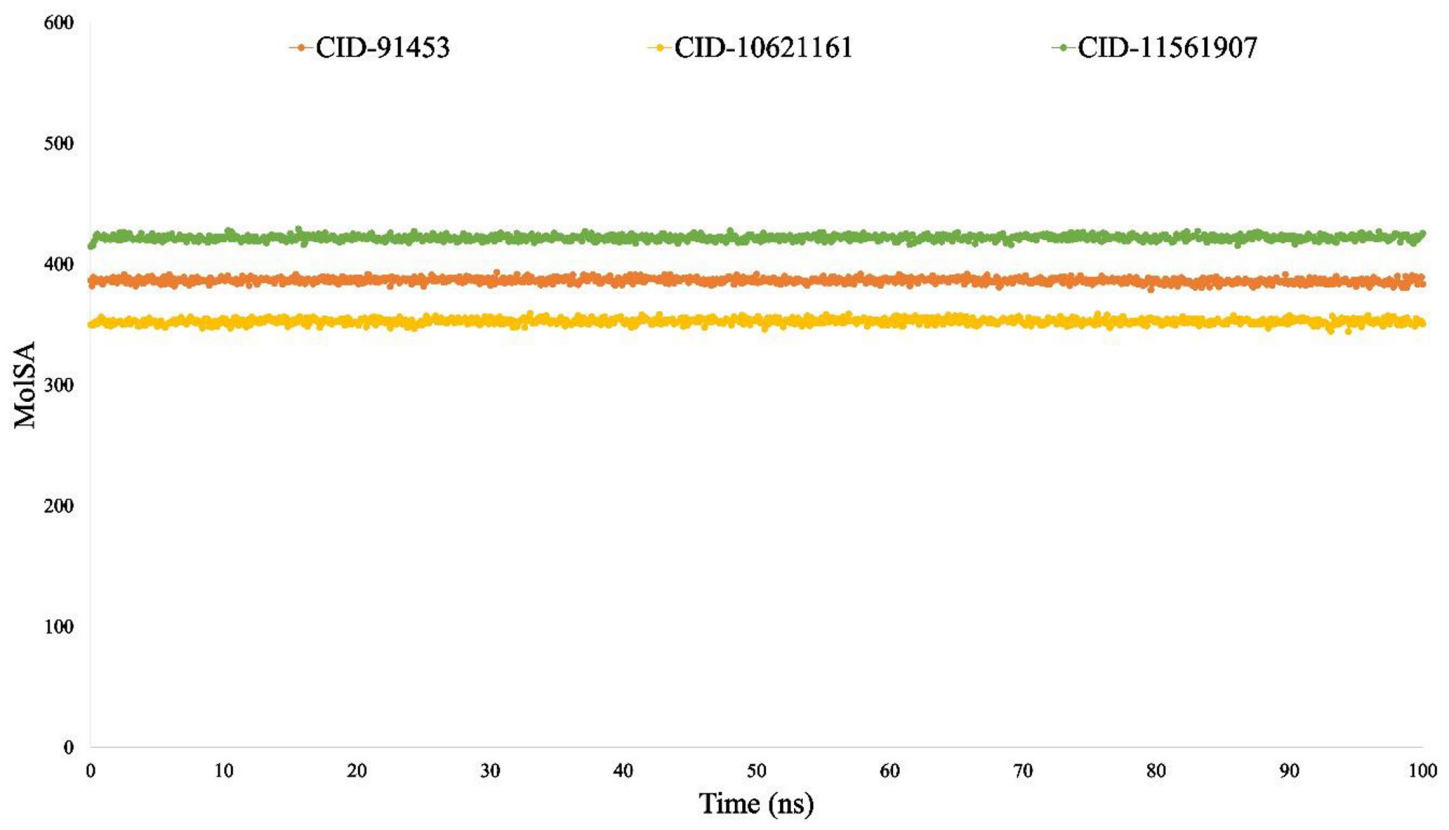
The molecular surface area (MoLSA) of the protein–ligand interaction was computed from the 100 ns simulation interaction diagram. The selected three ligands CID:91453 (Orange), CID: 10621161 (Yellow) and CID: 11561907 (Green) in connection with the selected protein.

**FIGURE 14:**
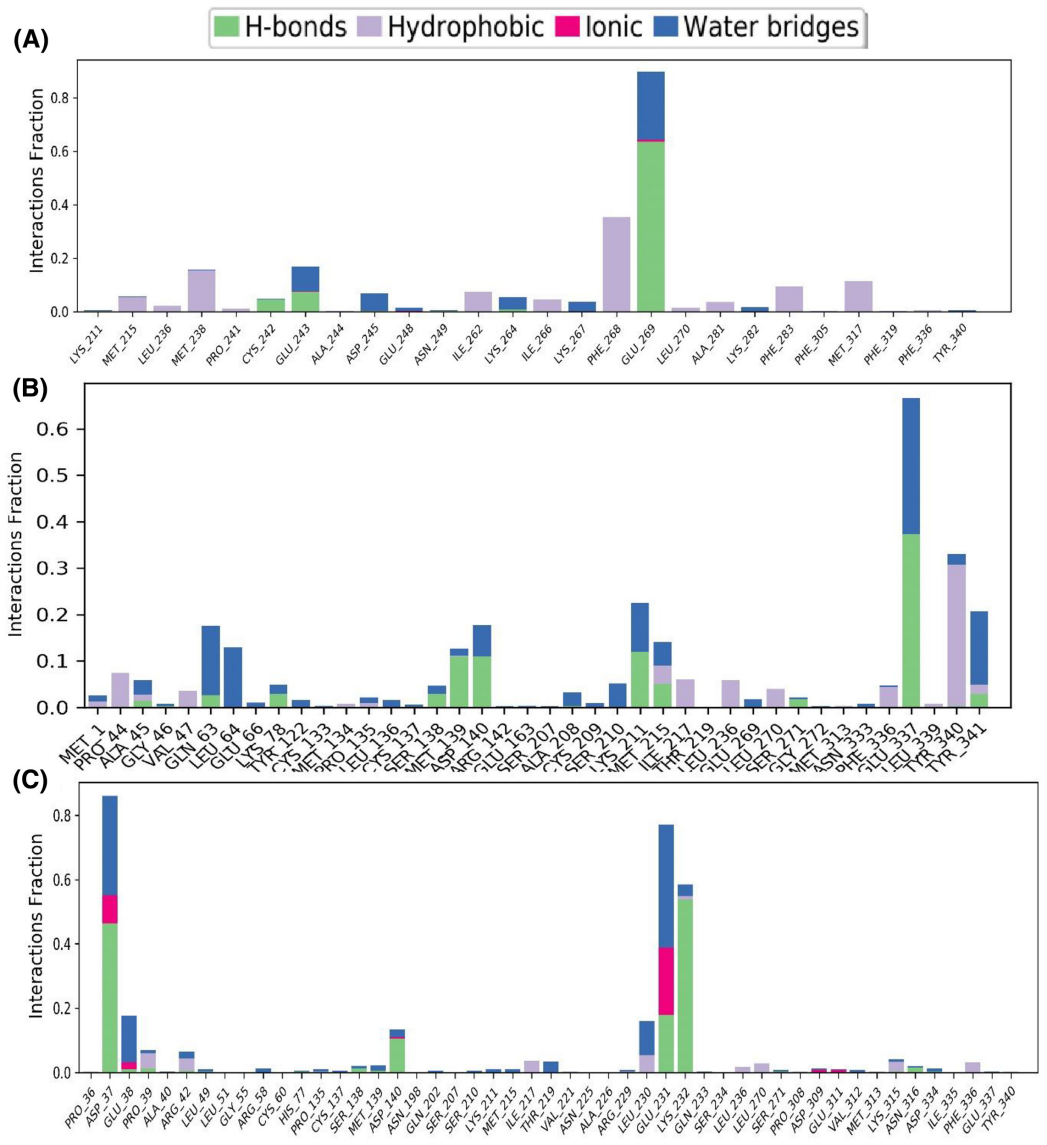
The stacked bar charts represent the protein–ligands interactions found during the 100 ns simulation. Herein, showing the interaction of selected three compounds, whereas (A) CID: 91453, (B) CID: 10621161, and (C) CID: 11561907 in complex with the P29279‐CCN2_HUMAN (Protein Accession: P29279), respectively.

The RMSD values of the complex system show the compounds ideal stability, whereas the RMSF values of the protein–ligand complex measure its compactness and represent the average fluctuation.^14^, ^119^ The minimum change in the protein structure is validated by the system's RMSD, which is calculated using the Cα atoms of the protein‐ligand complexes. The protein's fluctuation was also computed using the RMSF value, which verifies the complex system's minimal fluctuation and indicates the compounds stability with respect to the target protein. Many other metrics were examined to evaluate the complex's flexibility and stability, such as solvent‐accessible surface area (SASA), radius of gyration (Rg), number of hydrogen bonds and MoLSA. In this study, a 100‐nanosecond Molecular Dynamics Simulation (MDS) was carried out utilising the necessary physicochemical and physiological parameters, employing the Schrödinger software package (specifically, the Desmond application). Except for minor permissible fluctuations, cortistatin A, 13,16‐Epoxy‐25‐hydroxy‐17‐cheilanthen‐19,25‐olide and hecogenin displayed comparable similar RMSD and RMSF values with the protein P29279‐CCN2_HUMAN (Protein Accession: P29279) (Figures 9 and 10). The simulation results for various parameters, including the radius of gyration (Rg), hydrogen bond number, solvent accessible surface area (SASA) and MoLSA, were favourable when simulating with proteins linked with the development of HD (Figures 11, 12, 13, 14). This suggests the potential development of these compounds into medications for HD. Additionally, the Simulation Interaction Diagram (SID) was used in a 100‐nanosecond simulation to examine the intermolecular interactions that occur between proteins when they are complexed with certain ligands.^14^, ^120^, ^121^ The results demonstrated that throughout the simulation, all compounds formed numerous connections via ionic bonds, hydrophobic bonds, hydrogen bonds and water bridge bonds. Importantly, these connections persisted until the conclusion of the simulation, promoting the establishment of a stable binding with the targeted proteins.

Our findings showed that three potential drug candidates (cortistatin A, 13,16‐Epoxy‐25‐hydroxy‐17‐cheilanthen‐19,25‐olide and hecogenin) were identified based on low binding affinity and adherence to Lipinski's rule of five. Molecular dynamics simulations validated these findings. This research aids in designing effective HD therapeutics, with possibilities for additional wet lab inquiry.

## 5 CONCLUSION

This study aimed to identify important biomolecules and associated biochemical pathways utilizing integrative bioinformatics analysis. Ten DEGs, namely TPM1, EIF2S3, CCN2, ACTN1, ACTG2, CCN1, CSRP1, EIF1AX, BEX2 and TCEAL5, were identified as hub‐DEGs, presumably playing critical roles in HD development out of a total of 1743 DEGs. Enrichment analysis of these DEG through the gene ontology (GO) database unveiled significant functions such as Diphosphotransferase Activity, alpha‐N‐acetylgalactosaminide Alpha‐2,6‐Sialyltransferase Activity, Purine Ribonucleoside Triphosphate Binding, Protein Homodimerization Activity, ATP Binding, LRR Domain Binding, Ubiquitination‐Like Modification‐Dependent Protein Binding, Adenyl Ribonucleotide Binding, Translation Initiation Factor Activity and Mannosyl‐Oligosaccharide1,2‐Alpha‐Mannosidase. Potential regulatory biomarkers for both DEGs as well as hub‐DEGs, including projected regulatory TFs and miRNAs (such as hsa‐miR‐124‐3p as well as has‐miR‐26b‐5p), were found. By employing molecular docking alongside cross‐validation, we found three top‐ranked potential drug candidates (cortistatin A, 13,16‐Epoxy‐25‐hydroxy‐17‐cheilanthen‐19,25‐olide, Hecogenin) according to lowest binding affinity and followed Lipinski rule of five. By doing simulations with the molecular dynamics (MD) approach and consulting relevant literature, these results were confirmed. Consequently, the outcomes of this research might help greatly in designing an effective therapeutic method for HD. Future wet lab investigations may expand upon these study results.

## AUTHOR CONTRIBUTIONS

**Md Ridoy Hossain:** Conceptualization (equal); data curation (equal); formal analysis (equal); investigation (equal); methodology (equal); resources (equal); software (equal); validation (equal); visualization (equal); writing – original draft (equal). **Md. Mohaimenul Islam Tareq:** Data curation (equal); formal analysis (equal); investigation (equal); methodology (equal); resources (equal); software (equal); validation (equal); visualization (equal); writing – original draft (equal). **Partha Biswas:** Conceptualization (equal); data curation (equal); formal analysis (equal); investigation (equal); methodology (equal); resources (equal); software (equal); validation (equal); visualization (equal); writing – original draft (equal). **Sadia Jannat Tauhida:** Data curation (equal); formal analysis (equal); investigation (equal); methodology (equal); resources (equal); writing – original draft (equal). **Shabana Bibi:** Data curation (equal); formal analysis (equal); funding acquisition (equal); investigation (equal); methodology (equal); project administration (equal); resources (equal); software (equal); supervision (equal); validation (equal); visualization (equal); writing – review and editing (equal). **Md. Nazmul Hasan Zilani:** Data curation (equal); formal analysis (equal); investigation (equal); methodology (equal); project administration (equal); resources (equal); software (equal); supervision (equal); validation (equal); visualization (equal); writing – review and editing (equal). **Ghadeer M. Albadrani:** Data curation (equal); formal analysis (equal); funding acquisition (equal); investigation (equal); methodology (equal); project administration (equal); resources (equal); software (equal); visualization (equal); writing – review and editing (equal). **Muath Q. Al‐Ghadi:** Data curation (equal); formal analysis (equal); funding acquisition (equal); investigation (equal); methodology (equal); project administration (equal); resources (equal); software (equal); visualization (equal); writing – review and editing (equal). **Mohamed M. Abdel‐Daim:** Data curation (equal); formal analysis (equal); funding acquisition (equal); investigation (equal); methodology (equal); project administration (equal); resources (equal); software (equal); supervision (equal); visualization (equal); writing – review and editing (equal). **Md. Nazmul Hasan:** Data curation (equal); formal analysis (equal); funding acquisition (equal); investigation (equal); methodology (equal); project administration (equal); resources (equal); software (equal); supervision (equal); validation (equal); visualization (equal); writing – review and editing (equal).

## FUNDING INFORMATION

This research was funded by Princess Nourah bint Abdulrahman University Researchers Supporting Project number (PNURSP2024R30), Princess Nourah bint Abdulrahman University, Riyadh, Saudi Arabia and also funded by Researchers Supporting Project number (RSPD2024R811), King Saud University, Riyadh, Saudi Arabia.

## CONFLICT OF INTEREST STATEMENT

All authors declared that there is no conflict in others.

## Data Availability

The data are contained within the article.

## References

[1] McColgan P , Tabrizi SJ . Huntington's disease: a clinical review. Eur J Neurol. 2018;25(1):24‐34. doi:10.1111/ene.13413 28817209

[2] Moss DJH , Pardiñas AF , Langbehn D , et al. Identification of genetic variants associated with Huntington's disease progression: a genome‐wide association study. Lancet Neurol. 2017;16(9):701‐711. 28642124 10.1016/S1474-4422(17)30161-8

[3] van Hagen M , Piebes DGE , de Leeuw WC , et al. The dynamics of early‐state transcriptional changes and aggregate formation in a Huntington's disease cell model. BMC Genomics. 2017;18(1):373. doi:10.1186/s12864-017-3745-z 28499347 PMC5429582

[4] Xiang C , Cong S , Liang B , Cong S . Bioinformatic gene analysis for potential therapeutic targets of Huntington's disease in pre‐symptomatic and symptomatic stage. J Transl Med. 2020;18(1):388. doi:10.1186/s12967-020-02549-9 33054835 PMC7559361

[5] Khan H , Ullah H , Tundis R , et al. Dietary flavonoids in the management of Huntington's disease: mechanism and clinical perspective. eFood. 2020;1(1):38‐52.

[6] Ghosh R , Tabrizi SJJP . Clinical features of Huntington's disease. Polyglutamine Disorders. 2018;1049:1‐28. 10.1007/978-3-319-71779-1_1 29427096

[7] Myers RH , Sax DS , Schoenfeld M , et al. Late onset of Huntington's disease. J Neurol Neurosurg Psychiatry. 1985;48(6):530‐534. doi:10.1136/jnnp.48.6.530 3159849 PMC1028368

[8] Garcia TP , Marder K . Statistical approaches to longitudinal data analysis in neurodegenerative diseases: Huntington's disease as a model. Curr Neurol Neurosci Rep. 2017;17(2):14. doi:10.1007/s11910-017-0723-4 28229396 PMC5633048

[9] Shannon KM . Huntington's disease—clinical signs, symptoms, presymptomatic diagnosis, and diagnosis. Handb Clin Neurol. 2011;100:3‐13. doi:10.1016/b978-0-444-52014-2.00001-x 21496568

[10] Carbo M , Brandi V , Pascarella G , et al. Bioinformatics analysis of Ras homologue enriched in the striatum, a potential target for Huntington's disease therapy. Int J Mol Med. 2019;44(6):2223‐2233. doi:10.3892/ijmm.2019.4373 31638189 PMC6844632

[11] Azambuja MJ , Haddad MS , Radanovic M , Barbosa ER , Mansur LL . Semantic, phonologic, and verb fluency in Huntington's disease. Dement neuropsychol. 2007;1(4):381‐385. doi:10.1590/s1980-57642008dn10400009 29213415 PMC5619433

[12] Novak MJ , Tabrizi SJ . Huntington's disease: clinical presentation and treatment. Int Rev Neurobiol. 2011;98:297‐323. doi:10.1016/b978-0-12-381328-2.00013-4 21907093

[13] Harper PS . The epidemiology of Huntington's disease. Hum Genet. 1992;89(4):365‐376. doi:10.1007/bf00194305 1535611

[14] Khan DA , Adhikary T , Sultana MT , Toukir IA . A comprehensive identification of potential molecular targets and small drugs candidate for melanoma cancer using bioinformatics and network‐based screening approach. J Biomol Struct Dyn. 2023;1‐21. 10.1080/07391102.2023.2240409 37534476

[15] Davenport CB . Huntington's chorea in relation to heredity and eugenics. Proc Natl Acad Sci USA. 1915;1(5):283‐285. doi:10.1073/pnas.1.5.283 16575999 PMC1090803

[16] Rawlins MD , Wexler NS , Wexler AR , et al. The prevalence of Huntington's disease. Neuroepidemiology. 2016;46(2):144‐153. doi:10.1159/000443738 26824438

[17] Scrimgeour EM , Pfumojena JW . Huntington disease in black Zimbabwean families living near the Mozambique border. Am J Med Genet. 1992;44(6):762‐766. doi:10.1002/ajmg.1320440610 1481844

[18] Baig SS , Strong M , Quarrell OW . The global prevalence of Huntington's disease: a systematic review and discussion. Neurodegener Dis Manag. 2016;6(4):331‐343. doi:10.2217/nmt-2016-0008 27507223

[19] Bristy SA , Islam AH , Andalib KS , Khan U , Awal MA , Rahman MHJIMU . Determination of molecular signatures and pathways common to brain tissues of autism spectrum disorder: insights from comprehensive bioinformatics approach. Informat Med Unlocked. 2022;29:100871.

[20] Labbadia J , Morimoto RI . Huntington's disease: underlying molecular mechanisms and emerging concepts. Trends Biochem Sci. 2013;38(8):378‐385. doi:10.1016/j.tibs.2013.05.003 23768628 PMC3955166

[21] McFarland KN , Cha JH . Molecular biology of Huntington's disease. Handb Clin Neurol. 2011;100:25‐81. doi:10.1016/b978-0-444-52014-2.00003-3 21496570

[22] Dickey AS , La Spada AR . Therapy development in Huntington disease: from current strategies to emerging opportunities. Am J Med Genet A. 2018;176(4):842‐861. doi:10.1002/ajmg.a.38494 29218782 PMC5975251

[23] Bondi MW , Edmonds EC , Salmon DP . Alzheimer's disease: past, present, and future. J Int Neuropsychol Soc. 2017;23(9–10):818‐831. doi:10.1017/s135561771700100x 29198280 PMC5830188

[24] Zadel M , Maver A , Kovanda A , Peterlin B . DNA methylation profiles in whole blood of Huntington's disease patients. Front Neurol. 2018;9:655. doi:10.3389/fneur.2018.00655 30158895 PMC6104454

[25] Wang ZM , Dong XY , Cong SY . Bioinformatic analysis of a microRNA regulatory network in Huntington's disease. J Integr Neurosci. 2020;19(4):641‐650. doi:10.31083/j.jin.2020.04.203 33378838

[26] Dong X , Cong S . Identification of differentially expressed genes and regulatory relationships in Huntington's disease by bioinformatics analysis. Mol Med Rep. 2018;17(3):4317‐4326. doi:10.3892/mmr.2018.8410 29328442 PMC5802203

[27] Paulsen JS . Cognitive impairment in Huntington disease: diagnosis and treatment. Curr Neurol Neurosci Rep. 2011;11(5):474‐483. doi:10.1007/s11910-011-0215-x 21861097 PMC3628771

[28] Mredul MBR , Khan U , Rana HK , et al. Bioinformatics and system biology techniques to determine biomolecular signatures and pathways of prion disorder. Bioinform Biol Insights. 2022;16:11779322221145373. 36582393 10.1177/11779322221145373 PMC9793038

[29] Mastrokolias A , Ariyurek Y , Goeman JJ , et al. Huntington's disease biomarker progression profile identified by transcriptome sequencing in peripheral blood. Eur J Hum Genet. 2015;23(10):1349‐1356. doi:10.1038/ejhg.2014.281 25626709 PMC4592077

[30] Meem TM , Khan U , Mredul MBR , Awal MA , Rahman MH , Khan MS . A comprehensive bioinformatics approach to identify molecular signatures and key pathways for the Huntington disease. Bioinformat Biol Insights. 2023;17:11779322231210098. doi:10.1177/11779322231210098 PMC10683407 38033382

[31] Thomas EA . DNA methylation in Huntington's disease: implications for transgenerational effects. Neurosci Lett. 2016;625:34‐39. doi:10.1016/j.neulet.2015.10.060 26522374 PMC4864163

[32] Barrett T , Troup DB , Wilhite SE , et al. NCBI GEO: archive for high‐throughput functional genomic data. Nucleic Acids Res. 2009;37(suppl_1):D885‐D890. 18940857 10.1093/nar/gkn764 PMC2686538

[33] Chowdhury UN , Ahmad S , Islam MB , et al. System biology and bioinformatics pipeline to identify comorbidities risk association: neurodegenerative disorder case study. PLoS One. 2021;16(5):e0250660. doi:10.1371/journal.pone.0250660 33956862 PMC8101720

[34] Rahman MR , Islam T , Huq F , Quinn JM , Moni MAJ . Identification of molecular signatures and pathways common to blood cells and brain tissue of amyotrophic lateral sclerosis patients. Informat Med Unlocked. 2019;16:100193.

[35] Phipson B , Lee S , Majewski IJ , Alexander WS , Smyth GK . Robust HYPERPARAMETER ESTIMATION protects against hypervariable genes and improves power to detect differential expression. Ann Appl Stat. 2016;10(2):946‐963. doi:10.1214/16-aoas920 28367255 PMC5373812

[36] Benjamini Y , Hochberg YJ . Controlling the false discovery rate: a practical and powerful approach to multiple testing. J R Stat Soc B Methodol. 1995;57(1):289‐300.

[37] Calderoni S , Bellani M , Hardan AY , Muratori F , Brambilla P . Basal ganglia and restricted and repetitive behaviours in autism Spectrum disorders: current status and future perspectives. Epidemiol Psychiatr Sci. 2014;23(3):235‐238. doi:10.1017/s2045796014000171 24816251 PMC6998382

[38] Kuleshov MV , Jones MR , Rouillard AD , et al. Enrichr: a comprehensive gene set enrichment analysis web server 2016 update. Nucleic Acids Res. 2016;44(W1):W90‐W97. doi:10.1093/nar/gkw377 27141961 PMC4987924

[39] Harris MA , Clark J , Ireland A , et al. The gene ontology (GO) database and informatics resource. Nucleic Acids Res. 2004;32:D258‐D261. doi:10.1093/nar/gkh036 14681407 PMC308770

[40] Rahman MH , Peng S , Hu X , et al. Bioinformatics methodologies to identify interactions between type 2 diabetes and neurological comorbidities. IEEE Access. 2019;7:183948‐183970.

[41] Vivar JC , Pemu P , McPherson R , Ghosh S . Redundancy control in pathway databases (ReCiPa): an application for improving gene‐set enrichment analysis in omics studies and “big data” biology. OMICS. 2013;17(8):414‐422. doi:10.1089/omi.2012.0083 23758478 PMC3727566

[42] Waugh DF . Protein–protein interactions. Adv Protein Chem. 1954;9:325‐437. doi:10.1016/s0065-3233(08)60210-7 13217921

[43] Nooren IM , Thornton JM . Diversity of protein‐protein interactions. EMBO J. 2003;22(14):3486‐3492. doi:10.1093/emboj/cdg359 12853464 PMC165629

[44] Xia J , Gill EE , Hancock RE . Network Analyst for statistical, visual and network‐based meta‐analysis of gene expression data. Nat Protoc. 2015;10(6):823‐844. doi:10.1038/nprot.2015.052 25950236

[45] Smoot ME , Ono K , Ruscheinski J , Wang PL , Ideker T . Cytoscape 2.8: new features for data integration and network visualization. Bioinformatics (Oxford, England). 2011;27(3):431‐432. doi:10.1093/bioinformatics/btq675 21149340 PMC3031041

[46] Nogales‐Cadenas R , Carmona‐Saez P , Vazquez M , et al. GeneCodis: interpreting gene lists through enrichment analysis and integration of diverse biological information. Nucleic Acids Res. 2009;37:W317‐W322. doi:10.1093/nar/gkp416 19465387 PMC2703901

[47] Zhou G , Soufan O , Ewald J , Hancock REW , Basu N , Xia J . NetworkAnalyst 3.0: a visual analytics platform for comprehensive gene expression profiling and meta‐analysis. Nucleic Acids Res. 2019;47(W1):W234‐w241. doi:10.1093/nar/gkz240 30931480 PMC6602507

[48] Khan U , Rahman MH , Khan MS , Hossain MS , Billah MMJBR . Bioinformatics and network‐based approaches for determining pathways, signature molecules, and drug substances connected to genetic basis of schizophrenia etiology. Brain Res. 2022;1785:147889. 35339428 10.1016/j.brainres.2022.147889

[49] Jungo F , Bougueleret L , Xenarios I , Poux S . The UniProtKB/Swiss‐Prot Tox‐Prot program: a central hub of integrated venom protein data. Toxicon. 2012;60(4):551‐557. doi:10.1016/j.toxicon.2012.03.010 22465017 PMC3393831

[50] Jyothi P , Yellamma KJ . Molecular docking studies on the therapeutic targets of Alzheimer's disease (AChE and BChE) using natural bioactive alkaloids. Int J Pharm Pharm Sci. 2016;8(12):108‐112.

[51] Dallakyan S , Olson AJ . Small‐molecule library screening by docking with PyRx. Methods Mol Bol. 2015;1263:243‐250. 10.1007/978-1-4939-2269-7_19 25618350

[52] Daina A , Michielin O , Zoete V . SwissADME: a free web tool to evaluate pharmacokinetics, drug‐likeness and medicinal chemistry friendliness of small molecules. Sci Rep. 2017;7:42717. doi:10.1038/srep42717 28256516 PMC5335600

[53] Pires DE , Blundell TL , Ascher DB . pkCSM: Predicting small‐molecule pharmacokinetic and toxicity properties using graph‐based signatures. J Med Chem. 2015;58(9):4066‐4072. doi:10.1021/acs.jmedchem.5b00104 25860834 PMC4434528

[54] Lipinski CA , Lombardo F , Dominy BW , Feeney PJ . Experimental and computational approaches to estimate solubility and permeability in drug discovery and development settings. Adv Drug Deliv Rev. 2001;46(1–3):3‐26. doi:10.1016/s0169-409x(00)00129-0 11259830

[55] Banerjee P , Eckert AO , Schrey AK , Preissner R . ProTox‐II: a webserver for the prediction of toxicity of chemicals. Nucleic Acids Res. 2018;46(W1):W257‐W263. doi:10.1093/nar/gky318 29718510 PMC6031011

[56] Sahu N , Mishra S , Kesheri M , Kanchan S , Sinha RP . Identification of cyanobacteria‐based natural inhibitors against SARS‐CoV‐2 Druggable target ACE2 using molecular docking study, ADME and toxicity analysis. Indian J Clin Biochem. 2023;38(3):361‐373. doi:10.1007/s12291-022-01056-6 35812791 PMC9255548

[57] Azam MNK , Biswas P , Tareq MMI , et al. Identification of antidiabetic inhibitors from Allophylus villosus and Mycetia sinensis by targeting α‐glucosidase and PPAR‐γ: in‐vitro, in‐vivo, and computational evidence. Saudi Pharm J. 2024;32(1):101884. 38090733 10.1016/j.jsps.2023.101884 PMC10711519

[58] Sharma S , Sharma A , Gupta U . Molecular Docking studies on the Anti‐fungal activity of Allium sativum (Garlic) against Mucormycosis (black fungus) by BIOVIA discovery studio visualizer 21.1. 0.0. 2021.

[59] Wallace AC , Laskowski RA , Thornton JM . LIGPLOT: a program to generate schematic diagrams of protein‐ligand interactions. Protein Eng. 1995;8(2):127‐134. doi:10.1093/protein/8.2.127 7630882

[60] Roos K , Wu C , Damm W , et al. OPLS3e: extending force field coverage for drug‐like small molecules. J Chem Theory Comput. 2019;15(3):1863‐1874. doi:10.1021/acs.jctc.8b01026 30768902

[61] Kim M , Kim E , Lee S , Kim JS , Lee S . New method for constant‐ NPT molecular dynamics. J Phys Chem A. 2019;123(8):1689‐1699. doi:10.1021/acs.jpca.8b09082 30715880

[62] Basconi JE , Shirts MR . Effects of temperature control algorithms on transport properties and kinetics in molecular dynamics simulations. J Chem Theory Comput. 2013;9(7):2887‐2899. doi:10.1021/ct400109a 26583973

[63] Imon RR , Samad A , Alam R , et al. Computational formulation of a multiepitope vaccine unveils an exceptional prophylactic candidate against Merkel cell polyomavirus. Front Immunol. 2023;14:1160260. doi:10.3389/fimmu.2023.1160260 37441076 PMC10333698

[64] Singh SP , Konwar BK . Molecular docking studies of quercetin and its analogues against human inducible nitric oxide synthase. Springerplus. 2012;1(1):69. doi:10.1186/2193-1801-1-69 23556141 PMC3612180

[65] Umar AB , Uzairu A , Shallangwa GA , Uba SJSAS . Design of potential anti‐melanoma agents against SK‐MEL‐5 cell line using QSAR modeling and molecular docking methods. SN Applied Sciences. 2020;2(5):815.

[66] Šestić TL , Ajduković JJ , Marinović MA , Petri ET , Savić MP . In silico ADMET analysis of the A‐, B‐ and D‐modified androstane derivatives with potential anticancer effects. Steroids. 2023;189:109147. doi:10.1016/j.steroids.2022.109147 36410412

[67] Khaldan A , Bouamrane S , RE‐mM A , Sbai A , Bouachrine M , Lakhlifi TJ . Silico design of new α‐glucosidase inhibitors through 3D‐QSAR study, molecular docking modeling and ADMET analysis. Moroccan J Chem. 2022;10(1):22‐36.

[68] Ali SA , Hassan MI , Islam A , Ahmad F . A review of methods available to estimate solvent‐accessible surface areas of soluble proteins in the folded and unfolded states. Curr Protein Pept Sci. 2014;15(5):456‐476. doi:10.2174/1389203715666140327114232 24678666

[69] Shannon P , Markiel A , Ozier O , et al. Cytoscape: a software environment for integrated models of biomolecular interaction networks. Genome Res. 2003;13(11):2498‐2504. doi:10.1101/gr.1239303 14597658 PMC403769

[70] Junn E , Mouradian MM . MicroRNAs in neurodegenerative diseases and their therapeutic potential. Pharmacol Ther. 2012;133(2):142‐150. doi:10.1016/j.pharmthera.2011.10.002 22008259 PMC3268953

[71] Molasy M , Walczak A , Szaflik J , Szaflik JP , Majsterek I . MicroRNAs in glaucoma and neurodegenerative diseases. J Hum Genet. 2017;62(1):105‐112. doi:10.1038/jhg.2016.91 27412874

[72] Neueder A , Bates GP . A common gene expression signature in Huntington's disease patient brain regions. BMC Med Genet. 2014;7:60. doi:10.1186/s12920-014-0060-2 PMC4219025 25358814

[73] Dong X , Cong S . Bioinformatic analysis of microRNA expression in Huntington's disease. Mol Med Rep. 2018;18(3):2857‐2865. doi:10.3892/mmr.2018.9238 30015953 PMC6102687

[74] Träger U , Andre R , Lahiri N , et al. HTT‐lowering reverses Huntington's disease immune dysfunction caused by NFκB pathway dysregulation. Brain. 2014;137(Pt 3):819‐833. doi:10.1093/brain/awt355 24459107 PMC3983408

[75] Johnson R , Zuccato C , Belyaev ND , Guest DJ , Cattaneo E , Buckley NJ . A microRNA‐based gene dysregulation pathway in Huntington's disease. Neurobiol Dis. 2008;29(3):438‐445. doi:10.1016/j.nbd.2007.11.001 18082412

[76] Cha JH . Transcriptional signatures in Huntington's disease. Prog Neurobiol. 2007;83(4):228‐248. doi:10.1016/j.pneurobio.2007.03.004 17467140 PMC2449822

[77] Hwang YJ , Hyeon SJ , Kim Y , et al. Modulation of SETDB1 activity by APQ ameliorates heterochromatin condensation, motor function, and neuropathology in a Huntington's disease mouse model. J Enzyme Inhib Med Chem. 2021;36(1):856‐868. doi:10.1080/14756366.2021.1900160 33771089 PMC8008885

[78] Hwang HY , Kim TY , Szász MA , et al. Profiling the protein targets of unmodified bio‐active molecules with drug affinity responsive target stability and liquid chromatography/tandem mass spectrometry. Proteomics. 2020;20(9):e1900325. doi:10.1002/pmic.201900325 31926115

[79] Reynolds C , Damerell D , Jones S . ProtorP: a protein–protein interaction analysis server. Bioinformatics (Oxford, England). 2009;25(3):413‐414. doi:10.1093/bioinformatics/btn584 19001476

[80] Batada NN , Hurst LD , Tyers M . Evolutionary and physiological importance of hub proteins. PLoS Comput Biol. 2006;2(7):e88. doi:10.1371/journal.pcbi.0020088 16839197 PMC1500817

[81] Kundaje A , Meuleman W , Ernst J , et al. Integrative analysis of 111 reference human epigenomes. Nature. 2015;518(7539):317‐330. doi:10.1038/nature14248 25693563 PMC4530010

[82] Jin J , Cheng Y , Zhang Y , et al. Interrogation of brain miRNA and mRNA expression profiles reveals a molecular regulatory network that is perturbed by mutant huntingtin. J Neurochem. 2012;123(4):477‐490. doi:10.1111/j.1471-4159.2012.07925.x 22906125 PMC3472040

[83] Cardoso CM , de Jesus SF , de Souza MG , et al. High levels of ANXA2 are characteristic of malignant salivary gland tumors. J Oral Pathol Med. 2019;48(10):929‐934. 31325182 10.1111/jop.12932

[84] Shrivastava AN , Aperia A , Melki R , Triller A . Physico‐pathologic mechanisms involved in neurodegeneration: misfolded protein‐plasma membrane interactions. Neuron. 2017;95(1):33‐50. doi:10.1016/j.neuron.2017.05.026 28683268

[85] Nowak KJ , Ravenscroft G , Laing NG . Skeletal muscle α‐actin diseases (actinopathies): pathology and mechanisms. Acta Neuropathol. 2013;125(1):19‐32. doi:10.1007/s00401-012-1019-z 22825594

[86] Clarke NF , Kolski H , Dye DE , et al. Mutations in TPM3 are a common cause of congenital fiber type disproportion. Ann Neurol. 2008;63(3):329‐337. doi:10.1002/ana.21308 18300303

[87] Kotzaeridou U , Young‐Baird SK , Suckow V , et al. Novel pathogenic EIF2S3 missense variants causing clinically variable MEHMO syndrome with impaired eIF2γ translational function, and literature review. Clin Genet. 2020;98(5):507‐514. doi:10.1111/cge.13831 32799315 PMC7584729

[88] Borck G , Shin BS , Stiller B , et al. eIF2γ mutation that disrupts eIF2 complex integrity links intellectual disability to impaired translation initiation. Mol Cell. 2012;48(4):641‐646. doi:10.1016/j.molcel.2012.09.005 23063529 PMC3513554

[89] Moortgat S , Désir J , Benoit V , et al. Two novel EIF2S3 mutations associated with syndromic intellectual disability with severe microcephaly, growth retardation, and epilepsy. Am J Med Genet A. 2016;170(11):2927‐2933. doi:10.1002/ajmg.a.37792 27333055

[90] Skopkova M , Hennig F , Shin BS , et al. EIF2S3 mutations associated with severe X‐linked intellectual disability syndrome MEHMO. Hum Mutat. 2017;38(4):409‐425. doi:10.1002/humu.23170 28055140 PMC6267786

[91] Gregory LC , Ferreira CB , Young‐Baird SK , et al. Impaired EIF2S3 function associated with a novel phenotype of X‐linked hypopituitarism with glucose dysregulation. EBioMedicine. 2019;42:470‐480. doi:10.1016/j.ebiom.2019.03.013 30878599 PMC6492072

[92] Perbal B . CCN proteins: multifunctional signalling regulators. Lancet. 2004;363(9402):62‐64. doi:10.1016/s0140-6736(03)15172-0 14723997

[93] Welch MD , Howlett M , Halse HM , Greene WK , Kees UR . Novel CT domain‐encoding splice forms of CTGF/CCN2 are expressed in B‐lineage acute lymphoblastic leukaemia. Leuk Res. 2015;39(8):913‐920. doi:10.1016/j.leukres.2015.05.008 26138615

[94] Storey E , Beal MF . Neurochemical substrates of rigidity and chorea in Huntington's disease. Brain. 1993;116(Pt 5):1201‐1222. doi:10.1093/brain/116.5.1201 7693298

[95] Amsili S , Zer H , Hinderlich S , et al. UDP‐N‐acetylglucosamine 2‐epimerase/N‐acetylmannosamine kinase (GNE) binds to alpha‐actinin 1: novel pathways in skeletal muscle? PLoS One. 2008;3(6):e2477. doi:10.1371/journal.pone.0002477 18560563 PMC2423482

[96] Suzuki H , Takeuchi M , Sugiyama A , et al. Alternative splicing produces structural and functional changes in CUGBP2. BMC Biochem. 2012;13:6. doi:10.1186/1471-2091-13-6 22433174 PMC3368720

[97] Munguia E . Aberrant alternative splicing in skeletal muscle of R6/2 Huntington's disease mice. 2016.

[98] Chen S , Chen H , Yu C , et al. Long noncoding RNA myocardial infarction associated transcript promotes the development of thoracic aortic by targeting microRNA‐145 via the PI3K/Akt signaling pathway. J Cell Biochem. 2019;120:14405‐14413. 30989723 10.1002/jcb.28695

[99] Sehgal P , Narang S , Chawla D , et al. Systemic biomarkers of retinopathy of prematurity in preterm babies. Int Ophthalmol. 2023;43(5):1751‐1759. doi:10.1007/s10792-022-02576-z 36443542 PMC9707116

[100] Pan Y , Short JL , Newman SA , et al. Cognitive benefits of lithium chloride in APP/PS1 mice are associated with enhanced brain clearance of β‐amyloid. Brain Behav Immun. 2018;70:36‐47. doi:10.1016/j.bbi.2018.03.007 29545118

[101] Calvo L , Anta B , López‐Benito S , et al. Bex3 dimerization regulates NGF‐dependent neuronal survival and differentiation by enhancing trkA gene transcription. J Neurosci. 2015;35(18):7190‐7202. doi:10.1523/jneurosci.4646-14.2015 25948268 PMC6605261

[102] Jiang C , Wang JH , Yue F , Kuang S . The brain expressed x‐linked gene 1 (Bex1) regulates myoblast fusion. Dev Biol. 2016;409(1):16‐25. doi:10.1016/j.ydbio.2015.11.007 26586200 PMC4688182

[103] Jellinger KA . Basic mechanisms of neurodegeneration: a critical update. J Cell Mol Med. 2010;14(3):457‐487. doi:10.1111/j.1582-4934.2010.01010.x 20070435 PMC3823450

[104] Hijazi H , Reis LM , Pehlivan D , et al. TCEAL1 loss‐of‐function results in an X‐linked dominant neurodevelopmental syndrome and drives the neurological disease trait in Xq22.2 deletions. Am J Hum Genet. 2022;109(12):2270‐2282. doi:10.1016/j.ajhg.2022.10.007 36368327 PMC9748253

[105] Rahman MR , Islam T , Turanli B , et al. Network‐based approach to identify molecular signatures and therapeutic agents in Alzheimer's disease. Comput Biol Chem. 2019;78:431‐439. doi:10.1016/j.compbiolchem.2018.12.011 30606694

[106] Rahman MR , Islam T , Zaman T , et al. Identification of molecular signatures and pathways to identify novel therapeutic targets in Alzheimer's disease: insights from a systems biomedicine perspective. Genomics. 2020;112(2):1290‐1299. doi:10.1016/j.ygeno.2019.07.018 31377428

[107] Sevimoglu T , Arga KY . The role of protein interaction networks in systems biomedicine. Comput Struct Biotechnol J. 2014;11(18):22‐27. doi:10.1016/j.csbj.2014.08.008 25379140 PMC4212283

[108] Piccolo FM , Kastan NR , Haremaki T , et al. Role of YAP in early ectodermal specification and a Huntington's disease model of human neurulation. elife. 2022;11:e73075. doi:10.7554/eLife.73075 35451959 PMC9033270

[109] Ghafouri‐Fard S , Shoorei H , Bahroudi Z , Abak A , Majidpoor J , Taheri M . An update on the role of miR‐124 in the pathogenesis of human disorders. Biomed Pharmacother. 2021;135:111198. doi:10.1016/j.biopha.2020.111198 33412388

[110] Khan U , Khan MS . Prognostic value Estimation of BRIP1 in breast cancer by exploiting Transcriptomics data through bioinformatics approaches. Bioinform Biol Insights. 2021;15:11779322211055892. doi:10.1177/11779322211055892 34840500 PMC8619737

[111] Song J , Cho KJ , Oh Y , Lee JE . Let7a involves in neural stem cell differentiation relating with TLX level. Biochem Biophys Res Commun. 2015;462(4):396‐401. doi:10.1016/j.bbrc.2015.05.004 25976670

[112] Gao J , Liu QG . The role of miR‐26 in tumors and normal tissues (review). Oncol Lett. 2011;2(6):1019‐1023. doi:10.3892/ol.2011.413 22848262 PMC3406571

[113] Khan NH , Mir M , Qian L , et al. Skin cancer biology and barriers to treatment: recent applications of polymeric micro/nanostructures. J Adv Res. 2022;36:223‐247. doi:10.1016/j.jare.2021.06.014 35127174 PMC8799916

[114] Rahman MS , Zilani MNH , Islam MA , et al. In vivo neuropharmacological potential of gomphandra tetrandra (wall.) sleumer and in‐silico study against β‐amyloid precursor protein. PRO. 2021;9(8):1449.

[115] Aljahdali MO , Molla MHR , Ahammad F . Compounds identified from marine mangrove plant (*Avicennia alba*) as potential antiviral drug candidates against WDSV, an in‐Silico approach. Mar Drugs. 2021;19(5):253. doi:10.3390/md19050253 33925208 PMC8145693

[116] Bharadwaj S , Dubey A , Yadava U , Mishra SK , Kang SG , Dwivedi VD . Exploration of natural compounds with anti‐SARS‐CoV‐2 activity via inhibition of SARS‐CoV‐2 Mpro. Brief Bioinform. 2021;22(2):1361‐1377. doi:10.1093/bib/bbaa382 33406222 PMC7929395

[117] Lall RK , Adhami VM , Mukhtar H . Dietary flavonoid fisetin for cancer prevention and treatment. Mol Nutr Food Res. 2016;60(6):1396‐1405. doi:10.1002/mnfr.201600025 27059089 PMC6261287

[118] Paul P , Biswas P , Dey D , et al. Exhaustive plant profile of “dimocarpus longan lour” with significant phytomedicinal properties: a literature based‐review. PRO. 2021;9(10):1803.

[119] Aggarwal V , Tuli HS , Tania M , et al. Molecular mechanisms of action of epigallocatechin gallate in cancer: recent trends and advancement. Semin Cancer Biol. 2022;80:256‐275. doi:10.1016/j.semcancer.2020.05.011 32461153

[120] Durrant DE , Morrison DK . Targeting the Raf kinases in human cancer: the Raf dimer dilemma. Br J Cancer. 2018;118(1):3‐8. doi:10.1038/bjc.2017.399 29235562 PMC5765234

[121] Kanteti R , Mirzapoiazova T , Riehm JJ , et al. Focal adhesion kinase a potential therapeutic target for pancreatic cancer and malignant pleural mesothelioma. Cancer Biol Ther. 2018;19(4):316‐327. doi:10.1080/15384047.2017.1416937 29303405 PMC5902231

